# A hybrid feature weighted attention based deep learning approach for an intrusion detection system using the random forest algorithm

**DOI:** 10.1371/journal.pone.0302294

**Published:** 2024-05-23

**Authors:** Arshad Hashmi, Omar M. Barukab, Ahmad Hamza Osman

**Affiliations:** 1 Faculty of Computing and Information Technology in Rabigh (FCITR), Department of Information Systems, King Abdulaziz University, Jeddah, Saudi Arabia; 2 Faculty of Computing and Information Technology in Rabigh (FCITR), Department of Information Technology, King Abdulaziz University, Jeddah, Saudi Arabia; University of Alaska Anchorage, UNITED STATES

## Abstract

Due to the recent advances in the Internet and communication technologies, network systems and data have evolved rapidly. The emergence of new attacks jeopardizes network security and make it really challenging to detect intrusions. Multiple network attacks by an intruder are unavoidable. Our research targets the critical issue of class imbalance in intrusion detection, a reflection of the real-world scenario where legitimate network activities significantly out number malicious ones. This imbalance can adversely affect the learning process of predictive models, often resulting in high false-negative rates, a major concern in Intrusion Detection Systems (IDS). By focusing on datasets with this imbalance, we aim to develop and refine advanced algorithms and techniques, such as anomaly detection, cost-sensitive learning, and oversampling methods, to effectively handle such disparities. The primary goal is to create models that are highly sensitive to intrusions while minimizing false alarms, an essential aspect of effective IDS. This approach is not only practical for real-world applications but also enhances the theoretical understanding of managing class imbalance in machine learning. Our research, by addressing these significant challenges, is positioned to make substantial contributions to cybersecurity, providing valuable insights and applicable solutions in the fight against digital threats and ensuring robustness and relevance in IDS development. An intrusion detection system (IDS) checks network traffic for security, availability, and being non-shared. Despite the efforts of many researchers, contemporary IDSs still need to further improve detection accuracy, reduce false alarms, and detect new intrusions. The mean convolutional layer (MCL), feature-weighted attention (FWA) learning, a bidirectional long short-term memory (BILSTM) network, and the random forest algorithm are all parts of our unique hybrid model called MCL-FWA-BILSTM. The CNN-MCL layer for feature extraction receives data after preprocessing. After convolution, pooling, and flattening phases, feature vectors are obtained. The BI-LSTM and self-attention feature weights are used in the suggested method to mitigate the effects of class imbalance. The attention layer and the BI-LSTM features are concatenated to create mapped features before feeding them to the random forest algorithm for classification. Our methodology and model performance were validated using NSL-KDD and UNSW-NB-15, two widely available IDS datasets. The suggested model’s accuracies on binary and multi-class classification tasks using the NSL-KDD dataset are 99.67% and 99.88%, respectively. The model’s binary and multi-class classification accuracies on the UNSW-NB15 dataset are 99.56% and 99.45%, respectively. Further, we compared the suggested approach with other previous machine learning and deep learning models and found it to outperform them in detection rate, FPR, and F-score. For both binary and multiclass classifications, the proposed method reduces false positives while increasing the number of true positives. The model proficiently identifies diverse network intrusions on computer networks and accomplishes its intended purpose. The suggested model will be helpful in a variety of network security research fields and applications.

## I. Introduction

As Internet services are blossoming nowadays, efficient efforts are required for the identification of wicked, nefarious, or criminal activities over and around the network. Intrusion detection has been one of the best methods for finding anomalies. The network intrusion detection system (NIDS) monitors the network traffic for malicious traffic and policy violations and then generates alerts. Network security is the prime concern these days, and NIDS ensures it via pattern matching and classification of attacks into various classes; hence, this concern might be posed as a classification problem. An intrusion can be defined more appropriately in terms of integrity, confidentiality, and availability, which are the basic objectives of security [1]. When the breaching of these security objectives takes place, an intrusion is said to have occurred. An intrusion passes through. various stages, which are described as the probe stage, the exploitation stage, the action stage, and the masquerading stage. In the probe stage, the intruder can scan the victim’s system to find out potential flaws and collect information about the victim’s system. There arises the masquerading stage. In the probe stage, the intruder can scan the victim’s system to find out potential flaws and collect information about the victim’s system. There arises the requirement for an efficient and reliable network intrusion detection method that can distinguish between normal and anomalous activities with a minimum number of false alarms. Due to the demand of the hour, more and more researchers are getting associated with this field and are trying to develop more reliable methods [2]. The major focus of NIDS is to identify the correct type of an attack. Devising effective and precise detection methods is the need of the hour as the attacks are increasing day by day. According to a survey report [3], the most common attacks which have been identified until now include the attacks named Probe, User-to-root, Root-to-local, DoS, Brute-force, Structured Query Language (SQL) Injection, Malware, and phishing. These attacks interfere with the normal functioning of the network by sniffing sensitive information. Network traffic attacks have been classified into five types: DOS (Denial of service attacks), Normal, U2R (user-to-root attacks), Probe (Probing attacks), and R2l (root-to-local attacks). The fundamental problem is the identification of these attacks through the observation of peculiarly unanticipated malignant network traffic.

In an NIDS, machine learning algorithms are the most frequently used approach to classify network traffic as malicious or normal. Generally, classification approaches make use of shallow understanding and are based on feature learning and extraction. However, they fail to perform over a large amount of data, therefore giving high false alarm rates and low accuracy. There is a need to enhance the efficacy of classifiers in detecting malicious traffic. Several machine-learning technologies [4–8] have been employed for malicious communication identification in response to rising network traffic and the expansion of attack categories. Nonetheless, due to their limitations, traditional machine-learning approaches still need to be improved to meet the requirements of large-scale NIDS [9]. Deep learning (DL), a recent development in machine learning, has advanced in recent years in domains like image processing, natural language processing, and, most notably, network intrusion detection. Even with high-dimensional and unlabelled data, DL techniques attain significant accuracy levels in a short amount of time [10]. Many tedious tasks can be fulfilled with deep learning techniques. Due to the difficulty in categorizing attacks in intrusion detection, deep learning is now necessary [11]. Numerous advanced intrusion detection approaches based on deep learning have been proposed in NIDS, owing to its capacity for acquiring relevant features from voluminous data. Empirical research has demonstrated that deep learning can exhibit remarkable superiority compared to conventional approaches and can augment the effectiveness of attack identification processes [12]. According to research conducted on the KDD Cup’99 datasets [13], employing the LSTM-RNN technique resulted in a notably high detection rate. The study showed that, compared to other methods such as support vector machines (SVM), K-nearest neighbours (KNN), Bayes, probabilistic neural networks (PNNs), and several different neural network models, the performance of LSTM-RNN was superior. Torres et al. [14] investigated the efficacy of recurrent neural networks (RNN) for figuring out how network traffic behaves by modelling it as a sequence of states that evolve over time.

### 1. Motivation

Various deep learning methods, such as the DNN, CNN, LSTM, and RNN, are incorporated into an NIDS. This approach involves neural networks with significant depth for optimal functionality [15]. However, it does not utilize complete domain understanding of the network traffic. Inspired by these research works based on the deep learning approach, we presented our hybrid model called MCL-FWA-BILSTM to tackle the imbalance class issue utilizing the two techniques of the BI-LSTM-based semantic base feature weights and the self-attention-based feature weights. Both techniques enhanced domain knowledge acquisition and mitigated the effects of class imbalance. Class imbalance in intrusion detection is not just a statistical challenge rather it reflects the asymmetric nature of cybersecurity threats where legitimate network activities vastly outnumber malicious ones. In Section 2, a scholarly analysis of the various intrusion detection system using machine learning and deep learning techniques in the literature is presented.

### 2. Challenging issues

Deep learning techniques enabled the network intrusion detection systems (NIDS) to enhance significantly, producing formidable methods of identifying and mitigating cyber threats. However, despite that significant progress, the NIDSs still have a big problem in detecting some attacks, such as low-traffic attacks, mainly because of the issues of class imbalance occurring inside data sets [16]. In actual network attack conditions, most of the incoming and outgoing network data traffic is a minority part of it. This imbalance causes a significant learning environment distortion for deep learning models, which, in turn, results in much better performance on majority classes than accurately identifying the minority attack traffic. Such an imbalance has dire consequences: the rate of false positives is increased, thus affecting the effectiveness of NIDS, which also drops because of the many undetected cases. The core of the issue lies in the nature of modern NIDS datasets, which aim to mirror real-world traffic patterns. Realism is critical to enhancing the effective development of NIDS, but at the same time, it also presents one major challenge related to traffic under-representation. For example, deep learning models trained on such data may need to learn more about the characteristics of these less frequent attacks, due to which there is a possibility for them to be omitted or misclassified. This is not a technical problem only; instead, it bears critical vulnerabilities that attackers might use. Hence, the complete detection of any form of attack traffic becomes a paramount object for genuinely effective NIDS. Despite such a crucial need, there needs to be more literature and practice on how to address class imbalance in NIDS. Prior art to improve the performance of NIDS was either naive to the subtleties of class imbalance or needed to provide solutions that curb the problem to a great level. This is actually quite an overlooking surprise, given that an ideal intrusion detection system should be very good at recognizing every form of attack traffic, regardless of its frequency within the dataset. If, in a nutshell, deep learning is the fuel of advancement for NIDS, then class imbalance issues pose a curse. It hampers their potential to detect low-traffic attacks and affects their overall performance. Hence, the most crucial aspect for solving this problem in the next wave of NIDS development is an enhanced effort to refine the deep learning approaches or innovate new strategies that can handle imbalanced data well. Ultimately, the capability sought is one of balanced detection that has the highest possible accuracy along with the lowest possible rate of false alarms across the full range of cyber threat coverage, thereby ensuring the most comprehensive protection of networks.

### 3. Contribution

A novel hybrid deep learning model MCL-FWA-BILSTM is proposed. This model is unique due to its integration of multiple techniques: MCL (Mean Convolutional Layers), CNN (Convolutional Neural Network), BI-LSTM (Bidirectional Long Short-Term Memory), and self-attention mechanisms. The combination of these techniques is novel, particularly in the context of network IDS. Further, the proposed model aims to classify the intrusion into binary and multiclass attack categories. These attack categories are mentioned in Tables 2 and 3. The detailed contribution is described below:

- Attack Classification and Feature Extraction: The model classifies attacks into specific categories using a combination of CNN-MCL for average convolutional processing and BI-LSTM layers. Self-attention-based feature weighting is employed to enhance feature extraction, which is crucial for dealing with imbalanced datasets.
- Optimal Random Forest for Imbalanced Datasets: A significant innovation is the use of an optimal random forest algorithm that adjusts the weights of decision trees, giving more importance to minority classes. This approach addresses the class imbalance problem, a common challenge in network security datasets.
- BI-LSTM for Error Reduction and Feature Integration: The BI-LSTM network minimizes errors and aids in feature integration. It also helps in extracting semantic features, improving the model’s performance on imbalanced datasets.
- Attention Mechanism for Detailed Feature Extraction: The attention mechanism in the BI-LSTM model identifies key features in packet sequence data, enhancing anomaly detection capabilities.
- Performance Evaluation: The model’s performance was evaluated using two benchmark datasets (UNSW-NB15 and NSL-KDD), which are standard in IDS research. Our approach outperformed baseline models and previous research efforts, indicating its effectiveness in real-world scenarios

### 4. Novelty

The novelty lies in the hybrid approach combining deep learning with an ensemble technique for feature selection. This approach is particularly effective for handling imbalanced datasets and improving predictions for minority classes. The proposed model comprises three key components, starting with the Convolutional Neural Network (CNN), which is responsible for the non-linear mapping of features. This step is crucial as it enhances the representation of the data by capturing higher-level features through the network’s layers. Following the CNN, the Bidirectional Long Short-Term Memory (BI-LSTM) network works to improve feature representation by addressing the issue of overlapping features. The BI-LSTM processes data in both forward and reverse directions, ensuring that all temporal dependencies are captured, thus reducing redundancy in feature representation. These refined features are then optimized by an attention layer, which focuses on the most relevant features for the classification task by adjusting hyperparameters. The attention mechanism selectively emphasizes important features and diminishes the less important ones, which can lead to a reduction in noise within the feature set. Finally, the Random Forest classifier learns from the enhanced features. It is an ensemble method that can handle class imbalance by constructing multiple decision trees and aggregating their predictions, which generally leads to improved accuracy and robustness against overfitting. In summary, the model is designed to improve feature quality, reduce noise, and handle class imbalance, with the ultimate goal of enhancing accuracy in the classification of the dataset. In the present study, we assessed a novel technique on two widely-used benchmark datasets (UNSW-NB15 and NSL-KDD) and compared it with both baseline models and with previous research endeavours in this field. The rationale behind selecting these particular datasets was their prevalence as IDS datasets, encompassing contemporary network attacks that meet real-world attack criteria. Our hybrid approach yielded better results than most of the other approaches considered, according to our experimental findings. Our experiments confirmed that the proposed approach outperformed most other approaches available in the open literature.

The organization of this research article is as follows: The second section provides materials and methods. It contains related work of IDS’s existing algorithms and current research gaps. It describes in detail the suggested deep learning based hybrid model methodology in depth that contains (i) workflow of the proposed architecture and the overview; (ii) details of the Mean Convolutional Layer and the related algorithm, (iii) the description of Multiple Convolutional Layers, Pooling, and bidirectional-LSTM (BI-LSTM), (iv) the attention layer description and the bidirectional-attention (BI-ATT) algorithm details, (v) a description of the machine learning classifier used in the study (namely, the random forest (RF) classifier), and (vi) a criterion for evaluating the model’s performance. In Section three, experimental setup, dataset employed in the present work, the experimental results and their explanations are presented. In the Section 4, discussions are given, and proposed model performance is compared with the previous research work. Finally, the present study is concluded in Section 5.

## II. Materials and methods

### A. Related work

The present section reviews the most interesting and illustrative IDS research conducted in the last few years, especially on deep learning and machine learning. The application of deep learning technology in network intrusion detection systems (NIDS) has gained significant recognition owing to its exceptional ability to manage intricate and extensive datasets, as well as to extract the inherent features of traffic data. Consequently, such an application presents itself as a viable approach towards identifying security breaches. In recent years, many methods based on deep learning have been used to solve the intrusion classification problem. Following are few recent research works done in the field of ML and DL techniques and finally we present a summarized Table 1, in which we talk briefly about the DL-based approaches that have been developed recently and are available in the open literature.

**Table 1:** Related studies on IDS using deep learning.

Ref.	Aim	Methodology	Limitation
[17]	To use deep learning to improve network intrusion detection systems	The technique creates worm mutants and artificial signatures for NIDS use. (a) Creating new polymorphic worm mutants (a) Creating synthetic signatures (c) The creation of synthetic data	The mutation technique needs constant upkeeping. For similarity comparison, the Levenshtein algorithm concentrates on a global comparison of just two strings.
[18]	Utilizing DL and hyperparameters optimization to tackle the categorization of an IDS	This study proposes an alternative method for choosing DL structure-based models by automating the HPO procedure using a mixture of random and grid search methods.	In the event of an unbalanced assault dataset, the algorithm needs to enhance its performance.
[19]	Examining supervised feature selection approaches for NIDS.	(a) Assessing recent datasets using a custom-designed Python-based technique. (b) Giving a summary of the most well-known FS methods used in intrusion detection. (c) Evaluating different experimental approaches, including time complexity, performance, and feature correlation.	The used dataset has two drawbacks: firstly, the traffic was only acquired in a tiny testbed; and second, there are only 49 characteristics, which need to be improved to evaluate the efficacy of feature selection algorithms.
[20]	To create a novel approach to detecting network intrusions depending on multiple-stage DL image recognition.	ResNet50: a pre-trained model used for NIDS employing multi-stage DL Image Recognition.	Oversampling can compromise the integrity of the primary information. If oversampling mitigation techniques are used, the algorithm could take more time to train the model. The inability to classify something properly may occur from losing important data because of sloppy sampling.
[21]	The research work aims to develop an ML-based NIDS suitable for software-defined networks.	As a component of NIDS inside the SDN controller, ML techniques are employed for traffic monitoring to identify hostile behaviour inside the network. Random Forest, Decision Tree, and XGBoost are some of the classical and modern tree-based ML approaches used in this demonstration of attack detection.	NIDS may have trouble catching all packets in a dynamic or expansive network. Consequently, it may miss an attack initiated during a period of heavy traffic.
[22]	To more effectively collect spatial and temporal data using the hybrid convolutional recurrent neural network (HCRNN)-based network intrusion detection system.	CRNN: A CRNN is used to build a DL-based hybrid ID configuration that forecasts and sorts malicious cyberattacks within the network. It keeps track of local features. RNN: The ID system’s efficiency and precision are improved by the recurrent neural network’s capacity to recollect temporal data.	Only a single ID database is used to evaluate the HCRNNIDSm, a potential drawback of described strategy.
[23]	To come up with an efficient way to choose and classify features for network intrusion detection on cloud computing.	Identifying and comparing the best intrusion detection features using an ensemble approach. To detect intrusions, an ensemble-based classification model uses a majority voting strategy.	The KDD Cup99 dataset contains redundant records, which would hinder the performance of the assessed systems.
[24]	To make a DL model for detecting network intrusions with uneven data.	ADASYN: Adaptive synthetic sampling to increase the size of samples from minority groups. DLNID: a framework for finding traffic problems. Bi-LSTM: to train the sequence-based feature network. Autoencoder: used to reduce the size of data sets with the aim of improving information fusion CNN: to learn more about the features.	NSL-KDD dataset has less no of attacks as compared to UNSW-NB15 dataset.

In their research, Dutta et al. [25] present a hybrid network anomaly detection system consisting of two stages that utilize a Classical Autoencoder (CAE) and a deep neural network (DNN) for feature engineering and classification. The performance of the proposed model is evaluated on the UNSW-NB-15 dataset, resulting in an accuracy rate of 91.29%. Despite the significant advancements made to enhance NIDSs’ predictive abilities, recent studies have shed light on packet sampling techniques’ influence on NIDS models [26]. These investigations revealed that even minute sampling rates such as 1/100 and 1/1000 can substantially reduce Machine Learning (ML)-based NIDS systems’ performance. Aljbali et al. [27] presented a technique for detecting anomalies based on a bidirectional short-term memory (Bi-LSTM) algorithm. Experimenting with the UNSWNB15 dataset demonstrated that this approach outperformed other deep learning and ML models in terms of precision, recall, F1 score, and accuracy. The authors in [28] present a DL wireless intrusion detection (WIDS) approach that utilizes a feed-forward deep neural network (FFDNN). The FFDNN-WIDS scheme is equipped with an Extra Trees wrapper-based feature extraction module to produce an optimal input subset for the classifier. This study evaluated the performance of the FFDNN on the UNSW-NB15 dataset using binary and multiclass classification problems. Results revealed that the FFDNN network achieved high test accuracies of 87.10% (2-way) and 77.16% (10-way) for the UNSW-NB15 dataset. Tang et al. [29] introduced a deep stacking network (DSN) model that integrated the outputs of multiple classifiers to enhance the accuracy of intrusion detection systems. These authors claimed that a fusion approach with four classifiers improved the classification performance, yielding an overall accuracy rate of 86.8%. To achieve the best feature selection for NSLK-KDD, the authors in ref. [15] investigated the integration of the Long Short-Term Memory (LSTM) and the Genetic Algorithm (GA) approaches. The experimental outcomes reveal that their devised approach obtained a commendable accuracy level of 93.88%. Notably, no information was provided on the detection rate in this investigation.

### B. Gaps in the previous research

This section outlines the several research gaps identified in the literature review. Earlier studies utilized dummy datasets for investigating class imbalance, but this increased the false information and, in real conditions, increased the error in the imbalanced class. In the literature, some studies on feature selection have decreased the amount of information and increased the number of false positives. Several studies have utilized efficient feature mapping but still, they need to map the backward and forward planes of features.

### C. Proposed methodology

**Fig 1:**
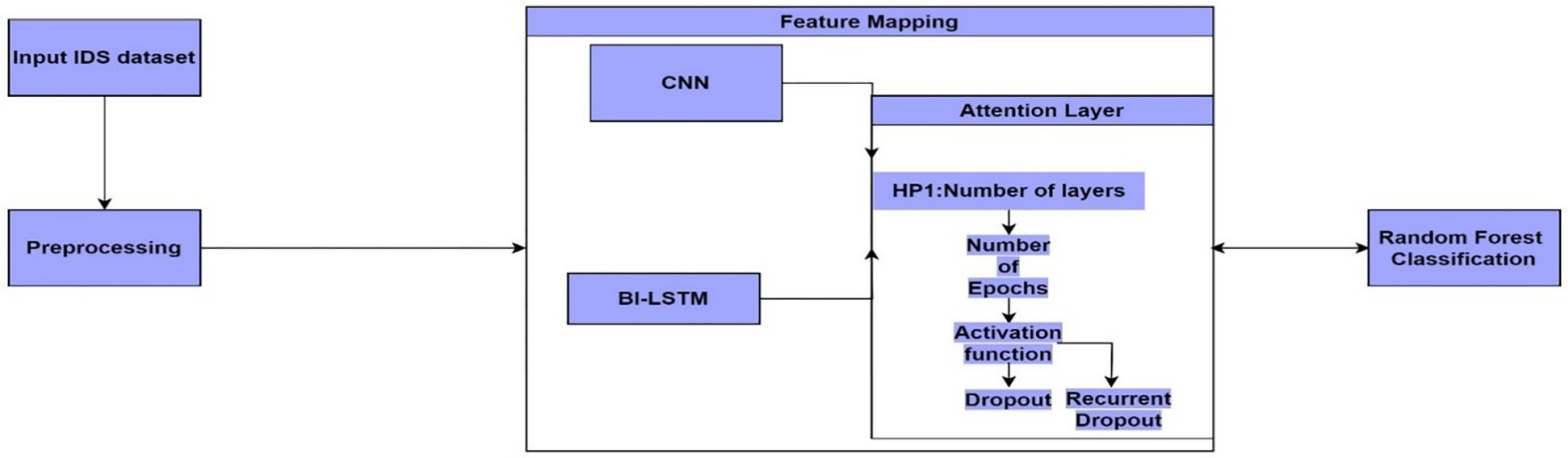
A diagram showing the workflow of the proposed architecture with hyperparameters to classify intrusions.

**Fig 2:**
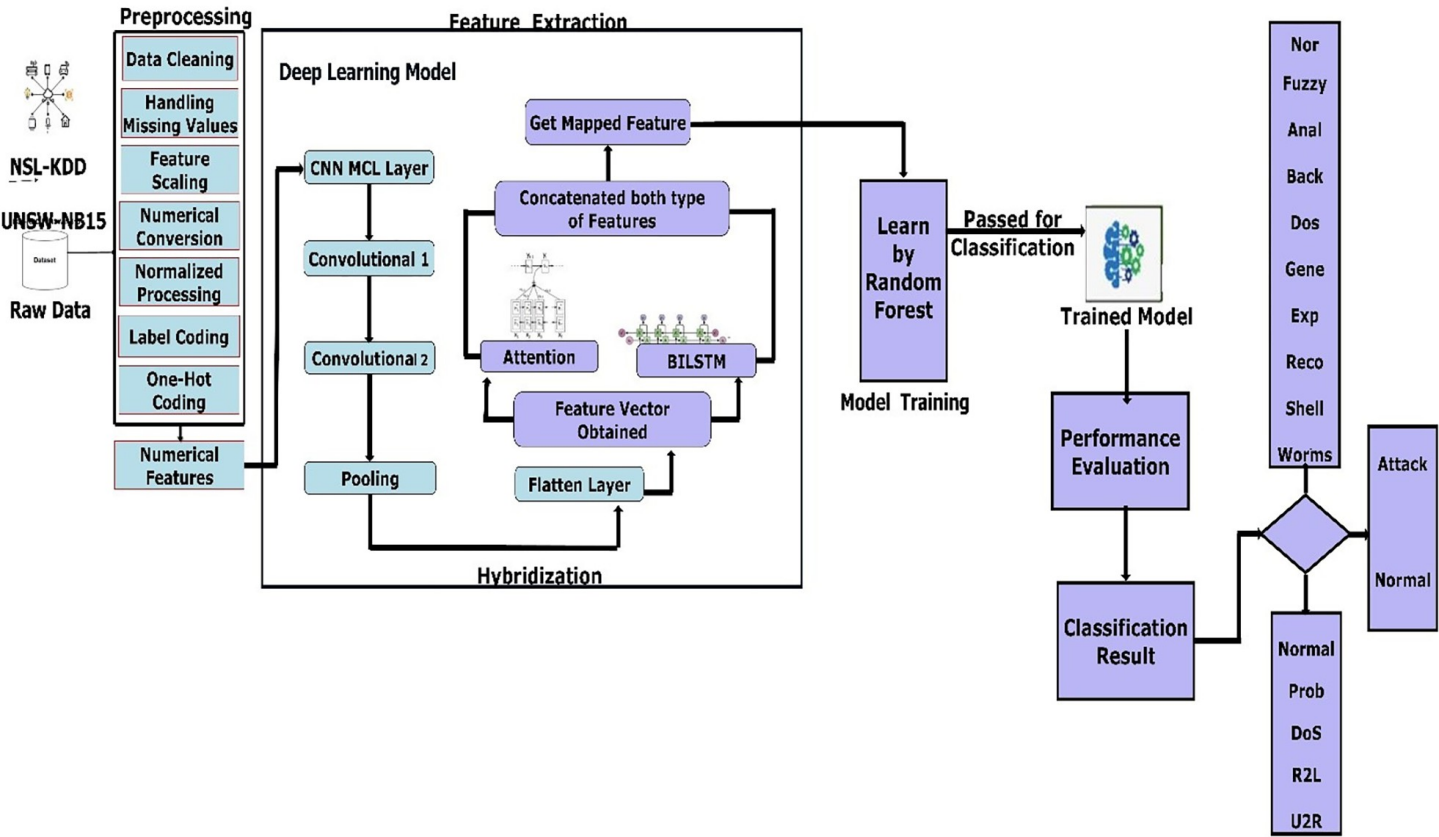
A diagram showing the workflow of the proposed novel hybrid- MCL-FWA-BILSTM based architecture to classify intrusions.

This study aimed to see how well the attention-based hybrid model works with deep learning techniques, specifically MCL, convolutional neural networks, and bidirectional long short-term memory networks, to classify network intrusions more accurately than with traditional techniques and classification using Random Forests. The NSL-KDD and UNSW-NB15 benchmark dataset served as a platform for experimentation. The workflow of the proposed architecture with hyperparameters to classify intrusions is shown in Fig 1 while the overall architecture of the hybrid MCL-FWA-BILSTM intrusion detection model is shown in Fig 2.

### D. Brief description of workflow of the proposed architecture

The Fig 1 depicts a flowchart of a machine learning pipeline designed for classification tasks, likely in the context of an Intrusion Detection System (IDS) given the label "Input IDS dataset". The process begins with the input IDS dataset, which then undergoes preprocessing to prepare the data for feature mapping. Feature mapping appears to be handled by two neural network architectures: Convolutional Neural Network (CNN) and a Bidirectional Long Short-Term Memory network (BI-LSTM). The CNN is typically used for capturing spatial hierarchies in data, while BI-LSTM is adept at processing sequences and capturing long-term dependencies in either direction of a sequence. Following feature extraction, an attention layer is applied, which can help the model to focus on more relevant parts of the data for the task at hand. The attention mechanism’s performance can be influenced by hyperparameters (HP1), such as the number of layers in the neural network, the number of training epochs, the choice of activation function, and the amount of dropout in recurrent connections. These hyperparameters are crucial for optimizing the performance of the model. Finally, the output of the attention layer feeds into a Random Forest classification algorithm. Random Forest is an ensemble learning method that operates by constructing a multitude of decision trees at training time and outputting the class that is the mode of the classes of the individual trees. This combination of neural networks with a traditional machine learning algorithm can leverage the strengths of both approaches to improve classification accuracy.

### E. Overview of the proposed architecture

Fig 2 depicts an overview of the suggested hybrid MCL-FWA-BILSTM (Mean Convolutional Layer-Feature Weighted Attention BILSTM) classification architecture for intrusions. In Section 3, Tables 2 and 3 detail the various types of attacks on datasets and the number of characteristics associated with each class label. The methodology of this study focuses on several NIDS components, and in the following paragraphs, we present brief details of crucial steps. In the first step, a dataset is used as input. To begin with, data preprocessing is required. The data preprocessing operation was carried out one by one on both the datasets NSL-KDD and UNSW-NB15 (see details in Section 3, Table 2 and 3). The primary operations at this stage include the missing value process, one hot coding, label coding, feature transformation, feature scaling, and feature normalization. In the next step, this work has a novel CNN-MCL layer designed for feature extraction before applying it to the attention mechanism. The CNN-MCL mapping is implemented using the mean convolutional layer (MCL), which represents average convolutional processing. Additional convolutional layers (Convolutional 1 and Convolutional 2) are utilized as feature extractors. Fig 2 also depicts a pooling layer and a flattening step following the convolution phase. The feature vector is obtained once the layer is flattened. Then, in the following steps, features from the attention layer and the BI-LSTM are concatenated to produce the mapped features. In the following steps, CNN-MCL features are assigned semantic basis feature weights and self-attention-based feature weights for enhancing the expression ability of the traffic features, which are then combined as mapped features to increase the class’s learning with fewer instances due to the semantic meaning learned from the integrated features and to make an efficient decision regarding the unbalanced class. Feature attention weights balance this procedure in normal classes. Finally, these mapped features are fed to random forests for classification, and the model’s performance is evaluated.

**Table 2:** Description of the NSL-KDD dataset attack categories.

Dataset		Total Instances Per Category	Description
Normal Category		77054	
Attack Category	DoS Denial of Service	53385	An intentional attempt to prevent access to data or services on a computer, network, or the Internet.
	Probe (Prob)	14077	This type of attack gathers information about how vulnerable the target system is, so that future attacks can be planned.
	R2L Remote to Local	3749	It is possible for an unauthorized person to get access to a remote system as a regular user or as a root by sending data packets to that machine over a network.
	U2R User to Root	252	By pretending to be real users, attackers can take advantage of security holes to gain root access to the system.
Total Instances		1,48,517	
Normal Instances		77054	
Attack instances		71463	

**Table 3:** Attack types and their description in the UNSW-NB15 dataset.

Category	Total Instances Per Category	Description	
Normal(Nor)	2218761	Natural transaction data	
Attack Category	Fuzzers (Fuzzy)	24246	Aiming to suspend a program or network by feeding it data produced randomly.
	Reconnaissance (Reco)	13987	Includes all strikes that can replicate information-gathering attacks.
	Shellcode (Shell)	1511	A tiny bit of code is used as the payload in software vulnerability exploitation.
	Analysis (Anal)	2677	It includes a variety of port scans, spam, and HTML file penetrations.
	Backdoors (Back)	2329	A method for gaining unauthorized access to a computer or its data by circumventing system security mechanisms.
	DoS	16353	A fraudulent action to render a server or network resource unavailable to users, typically by temporarily suspending or stopping the services of an Internet-connected host.
	Worms (Wrm)	174	To infect other systems, the attacker clones itself. Most of the time, it spreads through a computer network and gets into the target computer through some security holes.
	Generic (Gene)	215481	A method works against all block ciphers with a particular block and key size, regardless of how the block cipher is built.
	Exploits (Exp)	44525	The attacker is aware of a security flaw inside an operating system or piece of software and uses this information to take advantage of the flaw.
Attack Instances		321283	
Normal Instances		2218761	
Total Instances		2540044	

### F. Mean Convolutional Layer (CNN-MCL)

The suggested method employs CNN-based layers to completely separate anomalies from normal data. The data is used for learning the changes occurring from abnormal data. As normal and abnormal events are generally identical in characteristics, the CNN is used to detect abnormality variation. The traditional CNNs have been used to detect attacks and feature learning for flow content or matrix content, so that the classifier is connected with training data instead of learning data differences. Though, the suggested technique was to carefully evaluate the content in order to learn the anomalous traces.

We developed a novel hybrid architecture using mean convolutional layer (MCL), which is commonly used in intrusion detection tasks [30]. The suggested layer aims to completely learn prediction error filters in order to replicate these actions. Therefore, the active prediction error fields are affiliated with the feature maps as low-level abnormal trace quantities. The CNN-MCL is responsible for being aligned in the opposite direction of the CNN intended to initiate the IDS tasks. This serves as a data storage mechanism, as prediction errors do not typically involve flow content, and it also provides the CNN low-level IDS features. The more layers a CNN has, the more capable it is of learning higher-level features. The CNN-MCL is described using the following Eq (1), in which L signifies the Lth CNN-MCL, k represents the kth convolutional filter inside a layer, and the core value of a convolutional filter is characterized as (*C*_*x*_, *C*_*y*_). In addition, the CNN must learn prediction error filters by enforcing significant constraints actively.


$$F^{\left(L\right)}=Mean\left(W^{\left(L\right)}(C_{x},C_{y})\right)$$ (1)

$$\left\{ \begin{array}{l} w_{k}^{\left(L\right)}(x,y)=\frac{w_{k}^{\left(L\right)}\times F^{\left(L\right)}}{\Sigma w_{k}^{\left(L\right)}},(x,y)\neq (C_{x},C_{y}),\\ w_{k}^{\left(L\right)}(x,y)=-F^{\left(L\right)},\;(x,y)=(C_{x},C_{y}) \end{array}\right.$$ (2)

CNN-MCL predictions are recognized through a precise training phase. The filter weights $W_{k}^{\left(L\right)}$ are updated at every iteration using the Adam optimization algorithm during the following stage. By using CNN-MCL reinforcement, the updated filter weights are then integrated into the feasible set of prediction error filters, and a projection is executed on each training iteration. The central filter weight is set to a negative mean of middle values among all k filters inside the layer, and the remaining filter weights are normalized via Eq (2) above.

This methodology consists of two steps. The residual weights are partitioned by the sum of all filter weights, with the exception of the central value, after being multiplied by the mean value. The medians of all k filters are arranged to a negative mean value in layer L. Algorithm 1 below contains the pseudocode for this process:

Algorithm 1. CNN-MCL.

Input: Intrusion Dataset with features (F) and Labels

Output: Efficient Weight

Step 1. Initialize Random Weights (W) and  Set    i=1

Step 2. While ***i***≤***maximum*** iteration do

Do Feedforward pass

Update weight by Backpropagation using Adam

End While

$$Step3.\;SetFilterF^{\left(L\right)}=mean(tanh(F))$$ (3)

  mean of central points of all filters of layer L

Step 4. Update the weights of layers using

$$\begin{array}{c} W_{i}=(F^{\left(L\right)}*W_{i-1})/\sum_{i=0}^{N}W_{i}\\ i=i+1 \end{array}$$ (4)

Step 5. If Converge then exit

Step 6. Return W

Algorithm 1 gives efficient weights to features for improving the learning and its indirect impact on class imbalance. In the CNN-MCL algorithm, the iteration value depends on the training instances and the run-back propagation for reducing error. In the proposed approach shown in Fig 2, CNN-MCL filter Input Size N by 120, MCL layer N by 10 by 120, Conv1 N by 10 by 12 by 32, Conv2 5 by 5 by 16, flatten layer N by 56 after the weight is passed in the BI-ATT Algorithm 2 discussed in section J. Eq (3) in the CNN-MCL algorithm takes the mean of the values obtained by sigmoid mapping, with weights updated by Eq (4) and total weights also considered.

Algorithm 2. BI-ATT (Bidirectional Attention).

Input: Features and Weights

Output: Efficient mapped features

1. Input features F

2. $F_{B}<-tanh(W_{C}F_{t}+U_{C}F_{t-1}+b_{c})$

3.  $F_{B}<-tanh(W_{C}F_{t}+U_{C}F_{t-1}+b_{c})$

4.  ***F***_***A***_***<−by Eq*(1)**

5. ***F***_***AT***_**<−*by* Eq (2)**

6.  ***F***_***e***_***<−F***_***BL***_***ConcatF***_***AT***_

7.  ***ReturnF***_***e***_

### G. Multiple convolutional layers

The convolutional layer [31] is the most important component of a CNN. This layer applies multiple convolutional kernels to transform the feature maps (or input images) into new, unique feature maps. The deeper the network the more the layers might have a comprehensive range of vision and can capture global information [32]. So, as the number of convolutional layers increases, the scale of the convolutional features gradually gets bigger. A series of convolutional layers is used to learn higher-level prediction error features. As shown in Fig 2, each convolutional layer will learn the new representation of feature maps that the previous convolutional layer or lower-level features learned. The method described above shows how the Rectified Linear Unit (ReLu) activation function limits the range of data values at each stage of the network.

### H. Pooling layer

The CNN used a max-pooled size of 3x3 with a stride of 2. The max-pooling layer has the largest value in the sliding window’s local neighbourhood. This layer tries to reduce the number of dimensions in the feature maps. The max-pooling layer lowers the cost of training and the risk of overfitting. The pooling layers keep the most representative feature and aid in subsampling and improving accuracy.

### I. The Bidirectional Layer (BI-LSTM Layer)

During training, the BI-ISTM layer stores data in memory, sequentially represents long-distance correlation, and checks for correct gradient propagation. The LSTM model we use in our method is two-way, so it can be used to learn in both directions.

**Fig 3:**
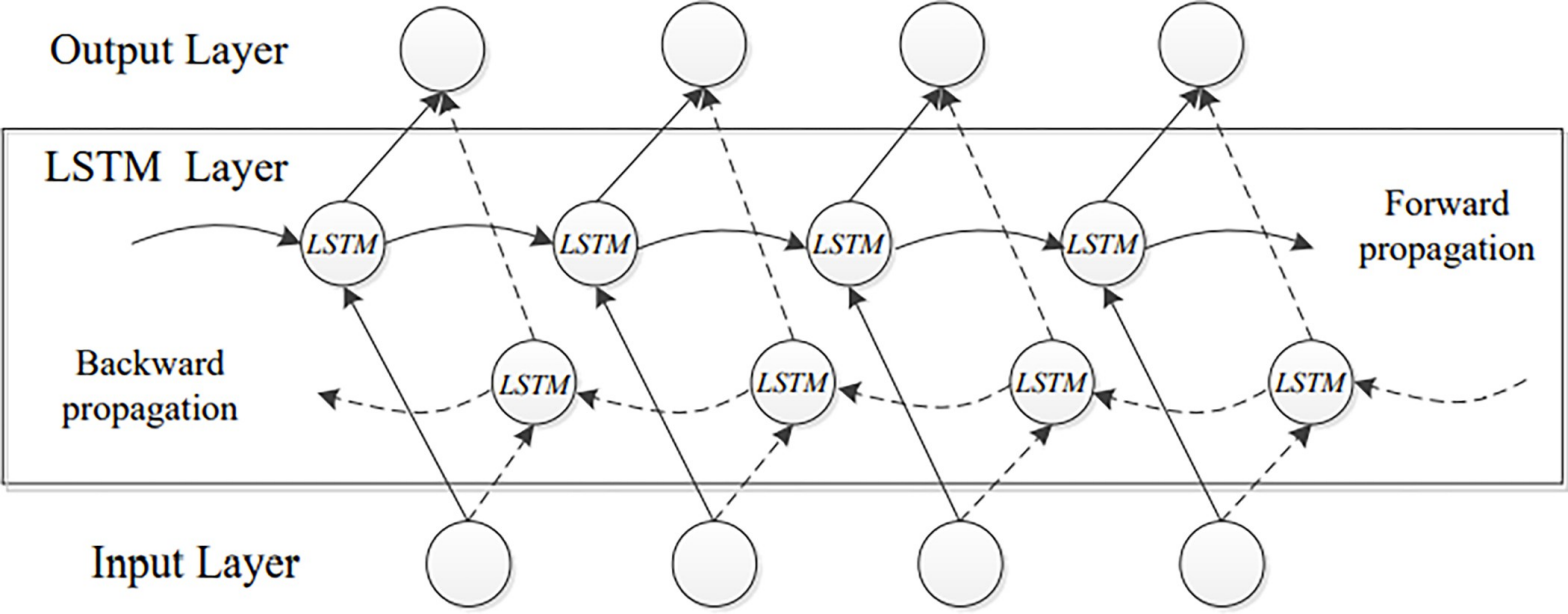
The architecture of the BI-LSTM model.

As stated in references [33,34] the BI-LSTM model is an improved form of the LSTM one. In order to extract coarse-grained features, the BI-LSTM model joins the forward LSTM model with the backward LSTM one. When new information is received, the LSTM model is trained to rewrite old content when new information is received. To perform the task first, it compares the contents of the innermost memory unit (Cu) via the input unit gate (***I***_***gt***_), the forget unit gate (***F***_***gt***_), and the output unit gate (***O***_***gt***_) [35]. Information sent into an LSTM network is evaluated for usefulness in light of applicable rules, and outliers are forgotten using the mechanism of the forget gate as indicated in Fig 3.

The hidden states of a BI-LSTM layer can be used in conjunction with an input arrangement ***z*** = ***z***_**0**_……..***z***_***t***_ at time t to yield the output sequence ***h*** = ***h***_**0**_……..***h***_***t***_. The result of employing this forget unit gate can be derived as

$$F_{gt}=sigmoid(W_{zF_{gt}}z_{t}+W_{hF_{gt}}h_{t-1}+b_{f})$$ (5)

Here ***h***_***t*−1**_ is the output of the hidden layer at time ***t***_**1**_ and ***z***_***t***_ is the input at time ***t***_**2**_ corresponding to the forget gate (***F***_***gt***_). The weights of the connections between the nodes are denoted by W, and the bias feature is denoted by b.

### J. Attention layer

This layer works for the attention mechanism used in the proposed work. Today, an attention mechanism is a powerful tool for identifying essential information and achieving excellent results [36,37]. The feature-based attention mechanism is adopted to fully capture the genuinely significant features of the representation of network traffic.

The BI-LSTM is capable of utilizing information from both sides. The self-attention mechanism improves the attention mechanism, which lessens reliance on outside information and more effectively captures the internal correlation of data or features. Our model can pay more attention to the crucial features of the network intrusion dataset by using the self-attention mechanism. This mechanism can affect both the source’s and the target’s internal components. As a result, it can increase the learning features’ efficiency during training [38]. The feature-weighted attention (FWA) technique is used to assign weight values to features. It is incorporated into classification models to aid in focusing on the most relevant features for classification and to minimize the overfitting issue. The FWA-BILSTM model concentrates on unique features that aid in identifying changes in the input data. This improves the classification performance of the FWA-BILSTM model and aids in determining the alteration in an input.

The basic concept of the attention mechanism is to extract and signify the most significant information in the data. The attention mechanism is an automatic weight allotting scheme. In intrusion detection, the role of the attention mechanism would be to calculate the effects of each unit of network traffic, mostly due to the preceding unit of network traffic. The attention value for every unit of network traffic is determined using the following equation

$$\alpha_{t}=\frac{\exp\left(u_{t}^{T}*u_{w}\right)}{\sum_{t}\exp\left(u_{t}^{T}*u_{w}\right)}$$ (6)

Where ***u***_***w***_ is indeed the weight matrix and ***u***_***t***_ is a matrix that acts as the implicit illustration of the CNN hidden state (***h***_***t***_) at time t.

The BI-LSTM model’s packet vectors ***h***_***t***_ are used in a nonlinear transformation to get its implicit representation ***μ***_***t***_ which can be written as

$$\mu_{t}=tanh(W_{w}h_{t}+b_{w})$$ (7)

In this case, ***W***_***w***_ denotes the weight matrix and ***b***_***w***_ denotes the bias. After the determination of the attention probability distribution value for every instant, the following formula is used to calculate the feature vector ***v*** containing the network traffic information:

$$\nu =\sum_{t}.\alpha_{t}*h_{t}$$ (8)

Finally, we can use the following function ***Y*** to obtain the predicted label y:

$$Y=Softmax(W_{w}*\nu +b_{\nu })$$ (9)

In Algorithm 2, we showed the non-linear mapping of features by the BI-LSTM RNN. This mapping improved class imbalance of IDs attacks, while the attention mechanism increased the reliability of features and the selection of an efficient mapping.

In Algorithm 2, F represents features and ***F***_***B***_ ***and F***_***BL***_ are weighted forward features and a combination of forwarded and backward feature mappings.

In $F_{B}<-tanh(W_{C}F_{t}+U_{C}F_{t-1}+b_{c})$, the Sigmoid Layer uses efficient weights that are obtained through the CNN-MCL approach.

The symbol ***W***_***c***_***F***_***t***_ denotes the initial weights obtained by the MCL layers and the CNN, and the symbol ***U***_***c***_***F***_***t*−1**_ denotes the previous layer non-linear mapping, while b represents the initial bias features.

In Algorithm 3, the proposed approach, which is called feature weighting by CNN-MCL starts with inputting weights Input to the BI-ATT algorithm, and, after feature mapping, it applies features or learning through the Random Forest algorithm by the bagging approach. After the learning phase, Algorithm 3 makes a classification model and analyzes performance metrics like accuracy, precision, recall, and F-score.

**Algorithm 3. Proposed Approach (Attention-Feature Weighting).**

Input: Dataset {features(F), Labels(X)}

Output: Classified Intrusion

1. Input Dataset and features

2. ***W***_***c***_**<−*CNN*−*MCL*(*F*)**

3. ***Fe***<−***BI***−***ATT***(***W***_***c***_,***F***)

4. ***C***<−***RandomForest***(***F***_***e***_,***X***)

5. ***AnalysisMetrics*<−*C*(*test*)**

### K. Random Forest (RF)

The RF algorithm can be described as an ensemble of classification trees that use the results of the decision tree (DT) model to make predictions. Each tree has one vote for the most frequent class in the input data. The trees are trained to grow together into a forest using a method called bagging or bootstrapping aggregation [39]. In the RF algorithm, the best predictor for each node is chosen randomly at the node level. By bagging on bootstrap sets of training data, it would be possible to make a lot of decision trees. The average or mean output value from the different decision trees (DTs) are used to make the final prediction of the RF algorithm. We used the RF algorithm to look at the behaviour of the intrusion from a different point of view because the RF algorithm builds multiple decision trees and combines them to make a more accurate and stable prediction. Several studies [40–42] have shown that the RF algorithm is better than other traditional classifiers at spotting strange traffic.

The loss function is generated in terms of the following objective function F:

$$F=\sum_{x=1}^{n}l(A_{x},{\hat{A}}_{x})+(\gamma T-(1-\gamma )T)+\frac{1}{2}\lambda \sum_{y=1}^{T}w_{y}^{2}$$ (10)

where, n denotes the given features with label instances and $\sum_{x=1}^{n}l(A_{x},{\hat{A}}_{x})$ denotes the training loss function, which fits the data into the L norm of the leaf node by the following equation:

$$L=(\gamma T-(1-\gamma )T)+\frac{1}{2}\lambda \sum_{y=1}^{t}w_{y}^{2}$$ (11)

All trees are assembled chronologically by using an additive learning process. Each newly added tree learns from its former tree and updates the prediction result by updating ${\hat{A}}^{k-1}$ at the kth iteration. In this way, the input data are classified into two types: (a) normal data and (b) malicious or anomalous data.

### L. Utilized metrics

Here, we use the most common metrics to measure how well the classifier model finds intrusions: precision, recall, false positive rate (FPR), false alarm rate (FAR), and F-score. The recall is also known as the Detection Rate (DR) in the intrusion detection problem. The detection rate (True Positive Fraction) generally represents the percentage of correctly classified malicious traffic. Similarly, the false positive rate (False Positive Fraction) shows the proportion of wrongly classified malicious traffic. Detection accuracy (DA) measures the system’s overall performance and demonstrates how well it can tell the difference between malicious and legitimate network traffic. In addition, the false alarm rate is the proportion of misclassified malicious and legitimate network traffic. The F-score is mainly utilized as a combination of Precision and Recall, which equals the Harmonic Mean of these two measures.

The Harmonic Mean is preferred to the Arithmetic Mean since the former punishes extreme values more severely. The F-score is used to evaluate the performance of every class of traffic. The performance of the proposed MCL-SA-BILSTM intrusion detection system is validated in terms of DR, Precision, FPR, and the F1-score. The definitions of these metrics are presented below:

RecallorDetectionRateDR=TP/(TP+FN) (12)

PrecisionPR=TP/(TP+FP) (13)

FalsepositiverateFPR=FP/(TN+FP) (14)

False Alarm Rate (FAR)—The false alarm rate represents the percentage of the normal and anomaly behaviours that are incorrectly classified.


FAR=FP+FN/2(TN+FP) (15)

DetectionAccuracyDA=TP+TN/(TP+TN+FN+FP) (16)

F‐scoreF=2*DR*PR/(DR+PR) (17)

**Fig 4:**
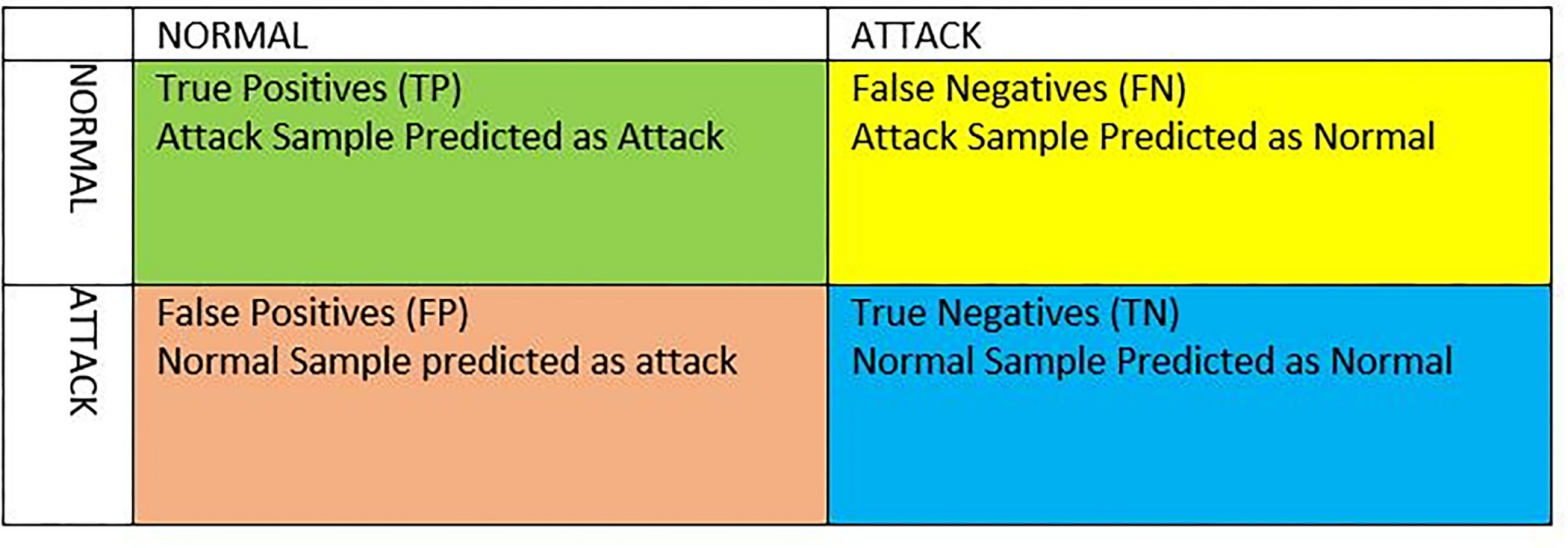
Confusion matrix related to IDS for performance evaluation.

The number of False Positives (FP) is the proportion of normal activities that are wrongly classified as an anomaly. The number of False Negatives (FN) is the proportion of anomalous behaviors that are incorrectly labelled as normal, whereas the number of True Negatives (TN) represents the proportion of normal behaviors that are accurately labelled as normal. Furthermore, the number of True Positives (TP) represents the proportion of anomalous behaviors that are accurately identified as an anomaly. The TP, TN, FP and FN are visualized in Fig 4.

The proposed approach consists of three main components. The first component is BILSTM, which is responsible for learning the sequence of features. Its length is set to 4 because LSTM units require four steps for error detection and correction, ensuring a fast learning rate by providing a value of 0.02. In our suggested approach, we set a hyperparameter Adam optimizer in the second stage, which involves hybridizing an attention layer and a mean CNN. The mean CNN consists of four layers with TANH and SoftMax activation functions. We next utilize a random forest algorithm, which employs boosting and bagging techniques, to tune the hyperparameters. The number of decision trees in the ensemble is determined through this process.

## III. Experimental setup

A computer with an Intel(R) Core(TM) i7-10875H processor running at 2.30 GHz, 32 GB of RAM, and an NVIDIA GeForce RTX 2070 GPU is used to carry out the experiments. The PC has a 64 bit MS Windows 11 operating system. Moreover, the proposed FWA-MCL-BILSTM model is implemented using Jupyter Notebook from Anaconda distribution, which is an open source. In addition, different libraries are used to implement the proposed system model. For example we have used several pandas libraries for the data analysis and manipulation of the data columns in the dataset. The pd.readcsv() function is used for reading the dataset. Using dataframe as df.isna.sum() and df.column.valuecounts() we analyzed the columns of the dataset. The df.info is used for datatype checking and df.describe() to get the statistical reports. In order to get the attack label train_data[’label’].value_counts() is used and for the distribution of attack classes train_data.label.value_counts() is used. We used pickle library to load and save the trained model. Further, the Numpy library is employed for converting the Pandas dataframe into multidimensional array objects to aid in performing calculations in faster mode. In order to normalize data, we used sklearn library StandardScaler(), and for multiclass label encoding we used LabelEncoder(), also for transformation we used LabelBinarizer from sklearn. In addition, this paper utilized TensorFlow, keras and pytorch library to implement out hybrid approach, such as the FWA,BILSTM, and CNN and MCL. Moreover, we have also used several built-in functions as well as user-defined functions in order to get enhanced performance. The experiments are carried out in two phases using the NSL-KDD dataset and the UNSW-NB15 dataset, as discussed in section III, and listed in Tables 2 and 3, respectively. The NSL-KDD dataset is used for the simulations in the first phase and the UNSW-NB15 dataset for the simulations in the second phase. The experiments conducted included both the binary and the multiclass classification tasks for both benchmark datasets.

### 1. Dataset

We assessed the suggested framework of the present study using two datasets, namely NSL-KDD and UNSW-NB15.

#### A. The NSL-KDD dataset

This research work employed the NSL-KDD dataset, which is available at http://www.unb.ca/research/iscx/dataset/iscx-NSL-KDD-dataset (accessed on November 25, 2023). This updated version of the KDD99 dataset mitigates issues concerning redundant data found in KDD99 and is widely utilized as a benchmark for IDS performance evaluation. The NSL-KDD includes training and test samples, encompassing 148517 traffic samples. The NSL-KDD dataset has 42 features, including nine basic Transmission Control Protocol (TCP) connection features, thirteen TCP connection content features, nine features based on the timing of network traffic, ten features based on the hosts involved in that traffic, and one label feature. These 42 features consist of one normal feature, 34 continuous features, four binary features, and three nominal features. The attacks considered can be divided into four groups based on their goals. These attacks include denial of service, probing, user-to-root, and attacker-to-intruder (R2L).

Additionally, binary classification involves two categories: normal and anomaly, while multiclassification divides classification labels into five categories—normal, dos, R2L, U2R, and probe—based on their specific features, among other dimensions. The attack categories are mentioned in Table 2.

#### B. UNSW-NB15 DATASET

This study used the UNSW-NB15 dataset, which is available at: https://research.unsw.edu.au/projects/unsw-nb15-dataset (as of October 20, 2023). The UNSW-NB15 dataset is an innovative dataset that was published in 2015. It contains modern nine attack types. It includes class labels for a total of 25,40,044 records. This dataset contains 49 features, including the class label and a wide range of normal and attacking activities. In total, there are 22,18,761 normal records, and 3,21,283 attacked records. The UNSW-NB15 dataset contains six distinct categories of features: flow features, basic features, content features, time features, labelled features, and additional generated features. The features between 36 and 40 are called general-purpose features, while features 41 to 47 are called connection features. The UNSW-NB15 dataset contains nine distinct attack categories: analysis, denial-of-service exploits, generics, reconnaissance, worms, and shellcode. Table 3 summarizes the attack types related to the UNSW-NB15 dataset including DoS, fuzzers, exploits, worms, shellcode, reconnaissance, generic analysis, and backdoors. Normal activities were assigned a value of zero, while the remaining nine attack types received a score of one. The former applies when no network intrusion occurs. By contrast, the latter describes situations where an Internet-based application is breached via port bypassing or unauthorized access to resources targeting security vulnerabilities.

### 2. Experimental results

In this section, we are going to analyze the performance of our MCL-FWA-BILSTM model in classifying network intrusion for the NSL-KDD and UNSW-NB15 datasets. Our assessment of the model outcome covers binary and multi-classification scenarios.

#### A. Multiclass experimental results for NSLKDD

We tested the suggested approach on the NSL-KDD dataset to prove its efficacy. Table 4 lists the confusion matrix for all attack classes in the NSL-KDD dataset while Table 5 shows additional evaluations, FPR, DR, F-score Precision and accuracy. With relative values of 76952, 53360, and 14000, the classes Normal, DoS, and Prob were identified with extreme precision. By contrast, the R2L (3610) and U2R (186) classes exhibited significantly less precision than the other classes.

**Table 4:** Confusion matrix of NSL-KDD on the proposed approach.

Classes	DOS	Normal	Prob	R2L	U2R
DOS	53360	8	8	0	0
Normal	30	76952	3	49	4
Prob	5	41	14000	0	0
R2L	2	228	2	3610	4
U2R	0	8	1	16	186

**Table 5:** Performance metrics of NSL-KDD dataset using MCL-FWA-BILSTM model.

Classes	FPR	DR	F-score	Precision	Accuracy
DoS	0.02	99.93	99.95	99.97	99.88
Normal	0.12	99.63	99.76	99.89	
Probe	0.03	99.9	99.79	99.67	
R2L	0.16	98.23	96	93.86	
U2R	0.02	95.88	91.85	88.15	

**Fig 5:**
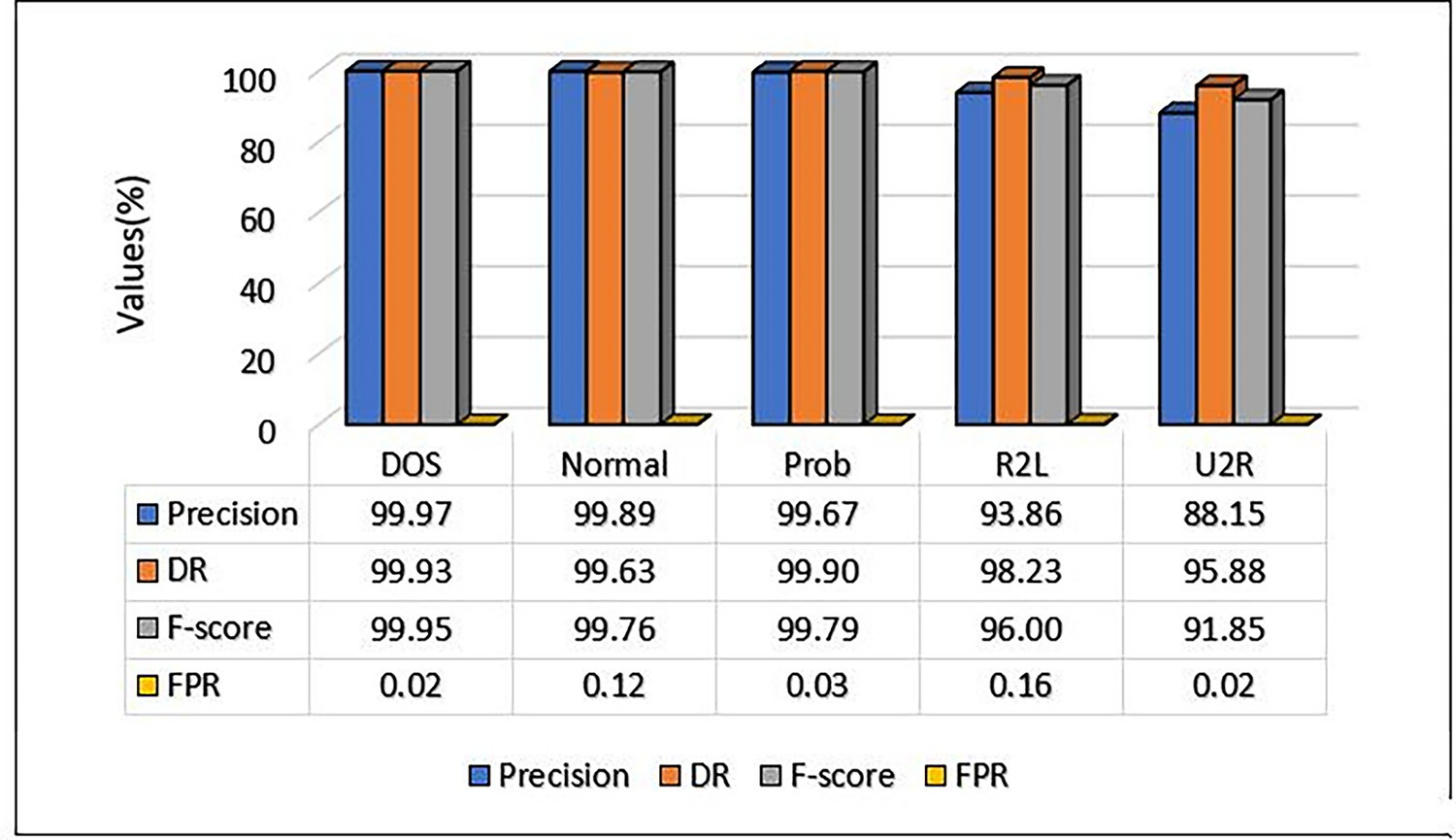
MCL-FWA-BILSTM model precision, DR, F-Score and FPR comparison on NSL-KDD dataset.

The proposed model’s performance details and the performance of a multi-class classification against various types of attacks are presented in Table 5 and visualised in Fig 5. For each of the four attack categories, the precision, DR, and F-score metrics are separately computed. For DoS attacks, the suggested model showed excellent performance in terms of precision, detection rate, and F-score, with a performance metric of almost 1.0 for all, whereas for probe and normal attacks, the suggested scheme accomplished a performance metric approximately equivalent to 1.0, with a difference of roughly 0.2. For R2L, the model achieved a performance metric in terms of precision that amounted to 93.86%. Other metrics obtained are 98.23% for the detection rate and 96% for the F-score. On the other hand, for U2R, the precision, DR, and F-score obtained are 88.15%, 95.88%, and 91.85% respectively. Further, the proposed model obtained an overall accuracy of 99.88% for the multiclass classification.

#### B. Binary classification results of NSL-KDD

The normal category has the value 0 and the remaining four categories (Dos, R2L, U2R, and Probe) are assigned the value 1. The assigned values are Normal = 0 and Attack = 1.

**Fig 6:**
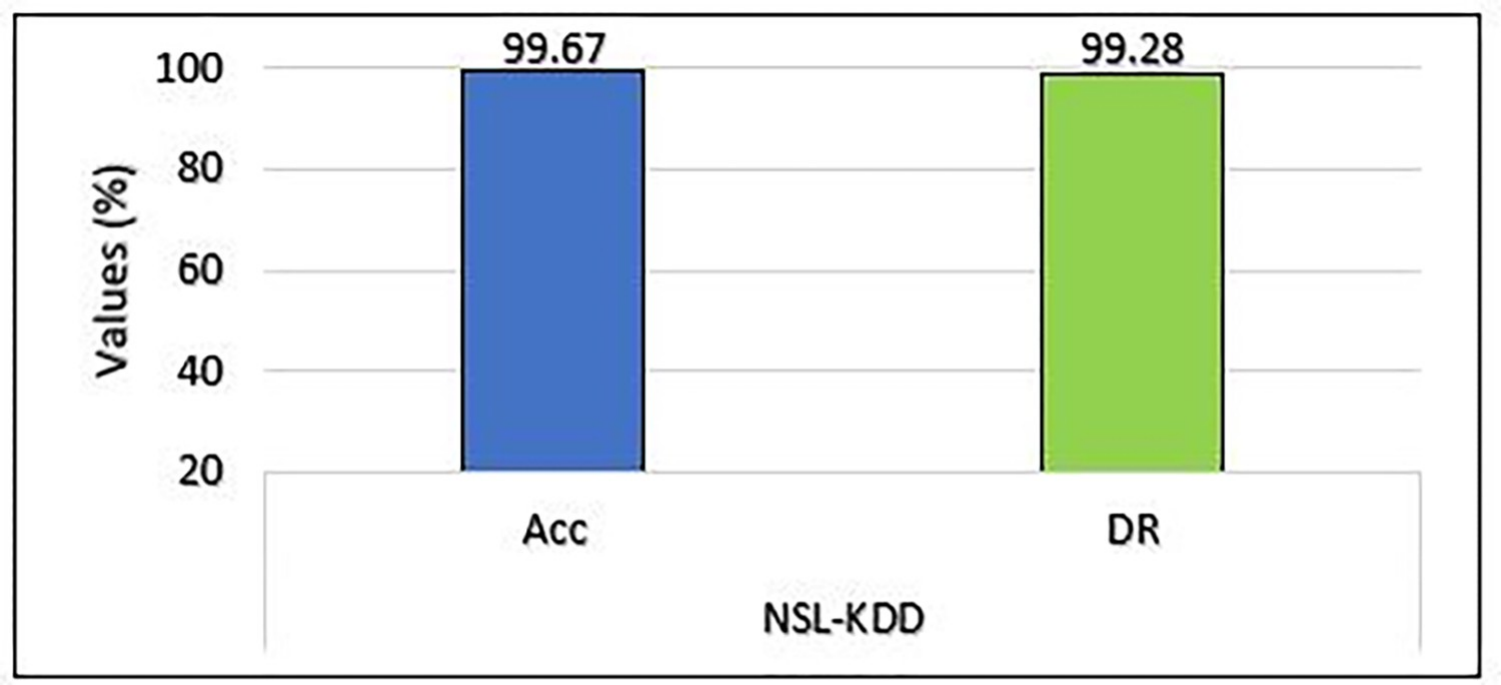
Binary classification accuracy and DR comparison on NSL-KDD.

The proposed model’s performance for binary classification is represented in Fig 6. The MCL-FWA-BILSTM model accuracy and detection rate are observed as 99.67% and 99.28, respectively for NSL-KDD dataset.

#### C. Multiclass experimental results for UNSW-NB15

In order to illustrate the proposed method’s efficacy, we also conducted the experiment on the UNSW-NB15 dataset. The confusion matrix for the suggested technique on UNSW-NB15 is shown in Table 6, and additional evaluations are included in Table 7. The precision, detection rate (DR), and F-score values are highest for the normal and generic classes, exceeding 98% in nearly all instances. By contrast, the Back and Worms classes have the lowest precision values, owing to their relatively low number of examples. Consequently, the imbalance in the UNSW-NB15 dataset could have been considered one of the possible explanations for the performance disparity between different classes.

**Table 6:** Confusion matrix of UNSW-NB-15 dataset using MCL-FWA-BILSTM approach.

Classes	Nor	Fuzzy	Anal.	Back.	DoS	Gene	Exp.	Reco.	Shell.	Worms.
Nor	94564	767	8	0	13	16	68	7	75	2
Fuzzy	495	20927	5	2	118	32	42	45	15	1
Anal.	27	121	530	1	3	0	17	1	0	0
Back.	9	49	0	422	49	2	88	19	15	1
Dos	81	357	5	2	9039	42	1200	21	33	2
Gene	42	70	0	2	87	57798	29	4	30	4
Exp	8	1219	1	7	900	9	56779	91	59	4
Reco	90	49	1	4	120	4	93	11470	31	0
Shell	34	158	0	3	14	36	22	11	1102	1
Worms	4	7	0	0	7	4	18	0	1	66

**Table 7:** Performance Metrics of the UNSW-NB15 dataset on the proposed approach.

Classes	Nor	Fuzzy	Anal.	Back.	Dos	Gene	Exp	Reco	Shell	Worms
Precision	99	96.5	96.5	64.35	83.33	99.54	96.11	96.11	79.88	61.68
DR	99.17	88.21	97.79	95.26	87.33	99.75	97.3	98.29	80.97	81.48
FPR	0.58	0.32	0.07	0.09	0.7	0.13	1.14	0.16	0.11	0.02
F-score	99.09	92.17	85.35	76.94	85.55	99.64	96.7	97.49	80.38	70.21
Acc.	99.45									

**Fig 7:**
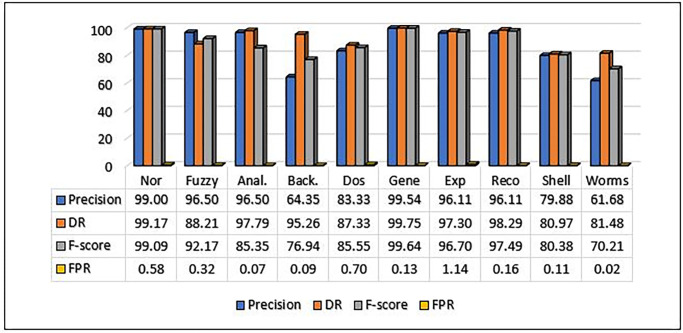
MCL-FWA-BILSTM model precision, DR, F-Score and FPR comparison on UNSW-NB15.

**Fig 8:**
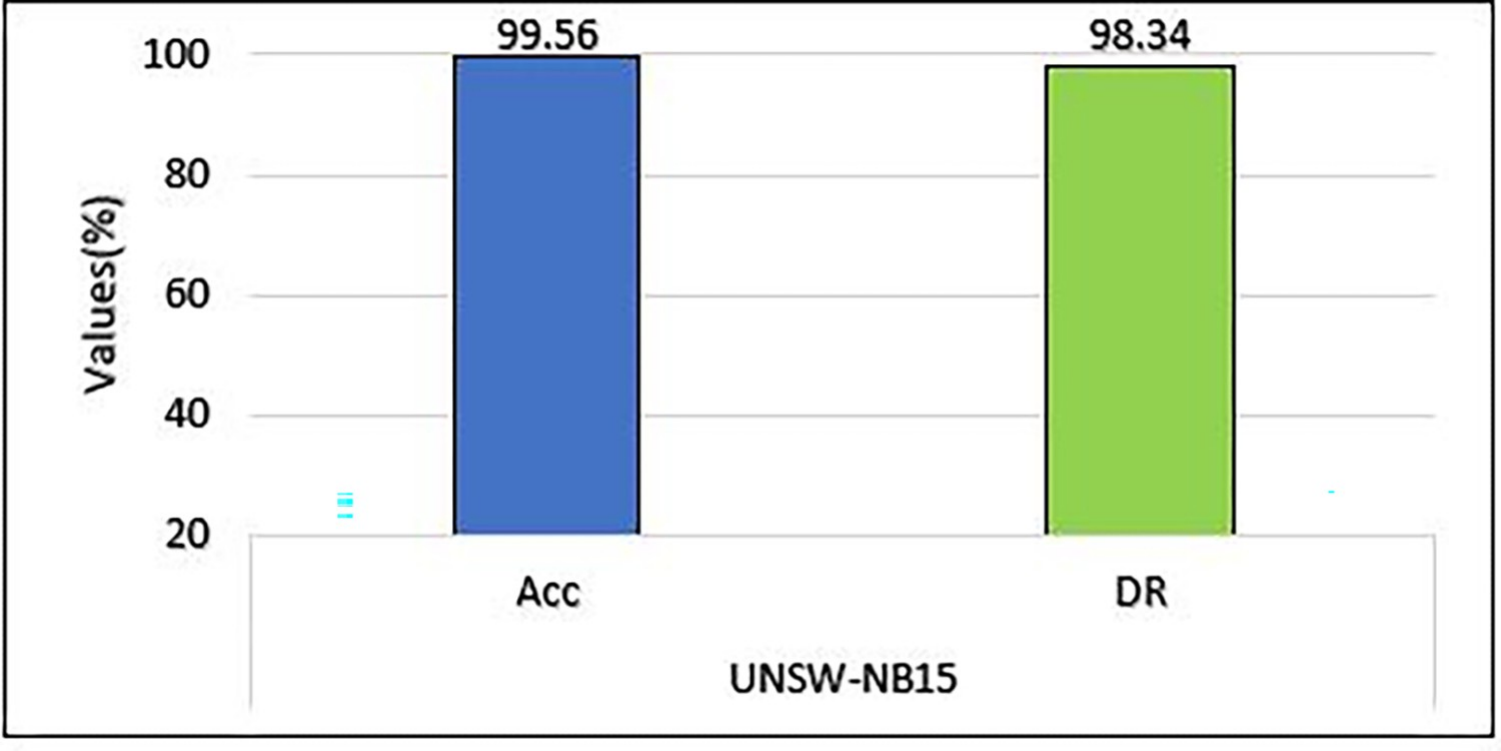
Comparison of accuracy and DR on UNSW-NB15.

**Fig 9:**
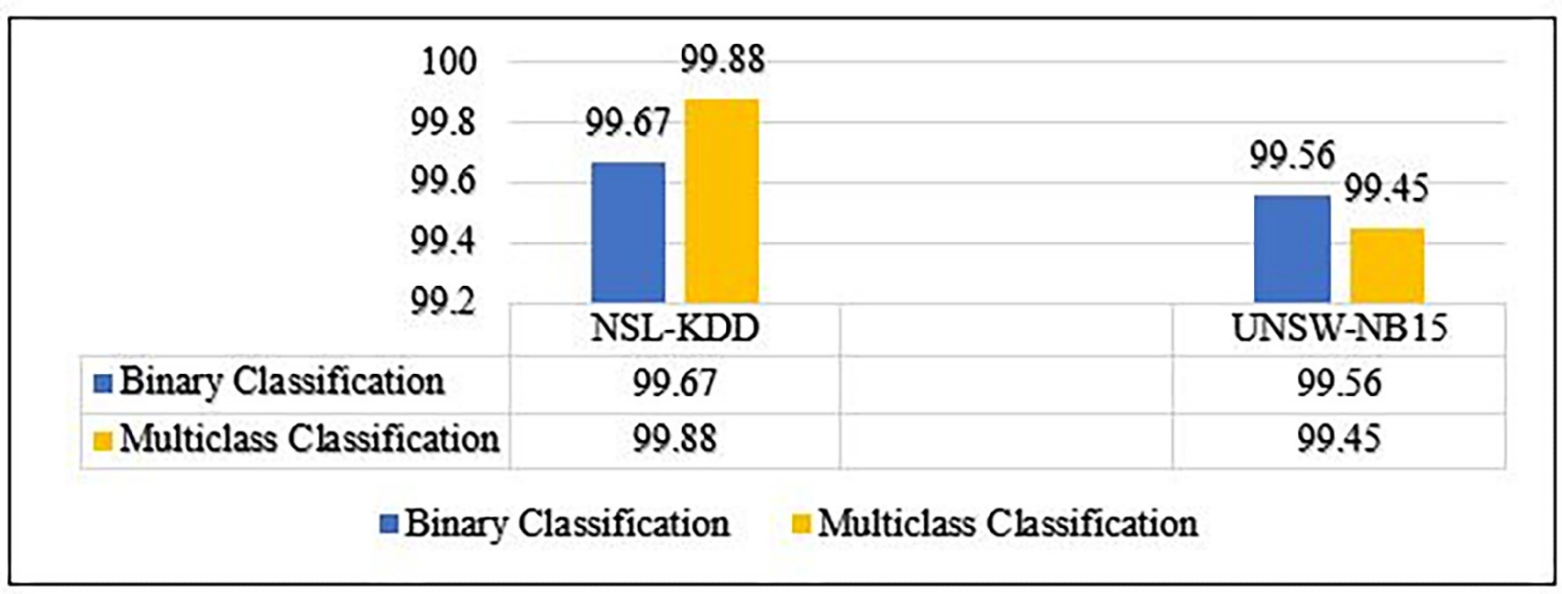
MCL-FWA-BILSTM binary and multiclass accuracy comparison.

The MCL-FWA-BILSTM model’s performance details, as well as the performance of the multi-class classification against various types of attacks, are shown in Table 7. The Precision, DR, and F-score are computed separately for each of the nine attack categories in addition to the normal class. The proposed MCL-FWA-BILSTM model achieved performance metric values close to 1.0 for all Exp, Reco, and Fuzzy attacks, with a difference of about 0.3. The proposed MCL-FWA-BILSTM model performed exceptionally well regarding the precision, DR, and F-score metrics for the Normal and Generic classes, with all values approaching 1.0. The precision for Back, DoS, Shell, and Worm is between 0.6 and 0.8, while DR is between 0.8 and 1.0, and the F-score is between 0.7 and 0.9.The Fig 7 represents the visualization of performance metrics of all the nine attack classes. The Fig 8, represents the accuracy of the binary classification and the Fig 9 represents the comparison of the binary and multiclass classification results.

## IV. Discussion

In this section, we discuss our achievements, and comparison with previous work. The Figs 10–18 and Tables 8–13 show the whole results of our investigation. Hence, we will carefully analyze each of them separately and comprehensively.

**Fig 10:**
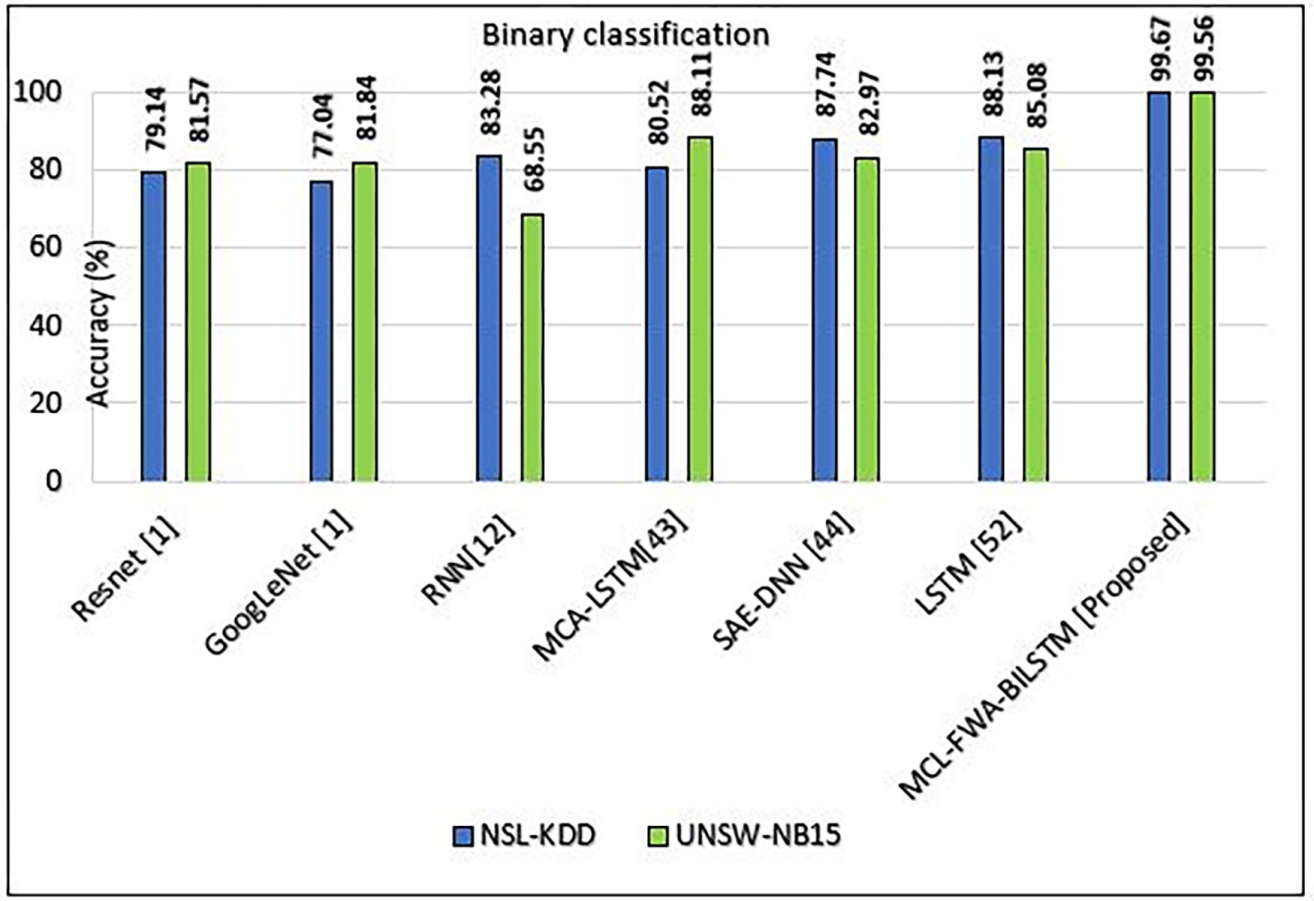
Comparison of accuracy of MCL-FWA-BILSTM model with existing models for binary classification on UNSW-NB15 and NSL-KDD.

**Fig 11:**
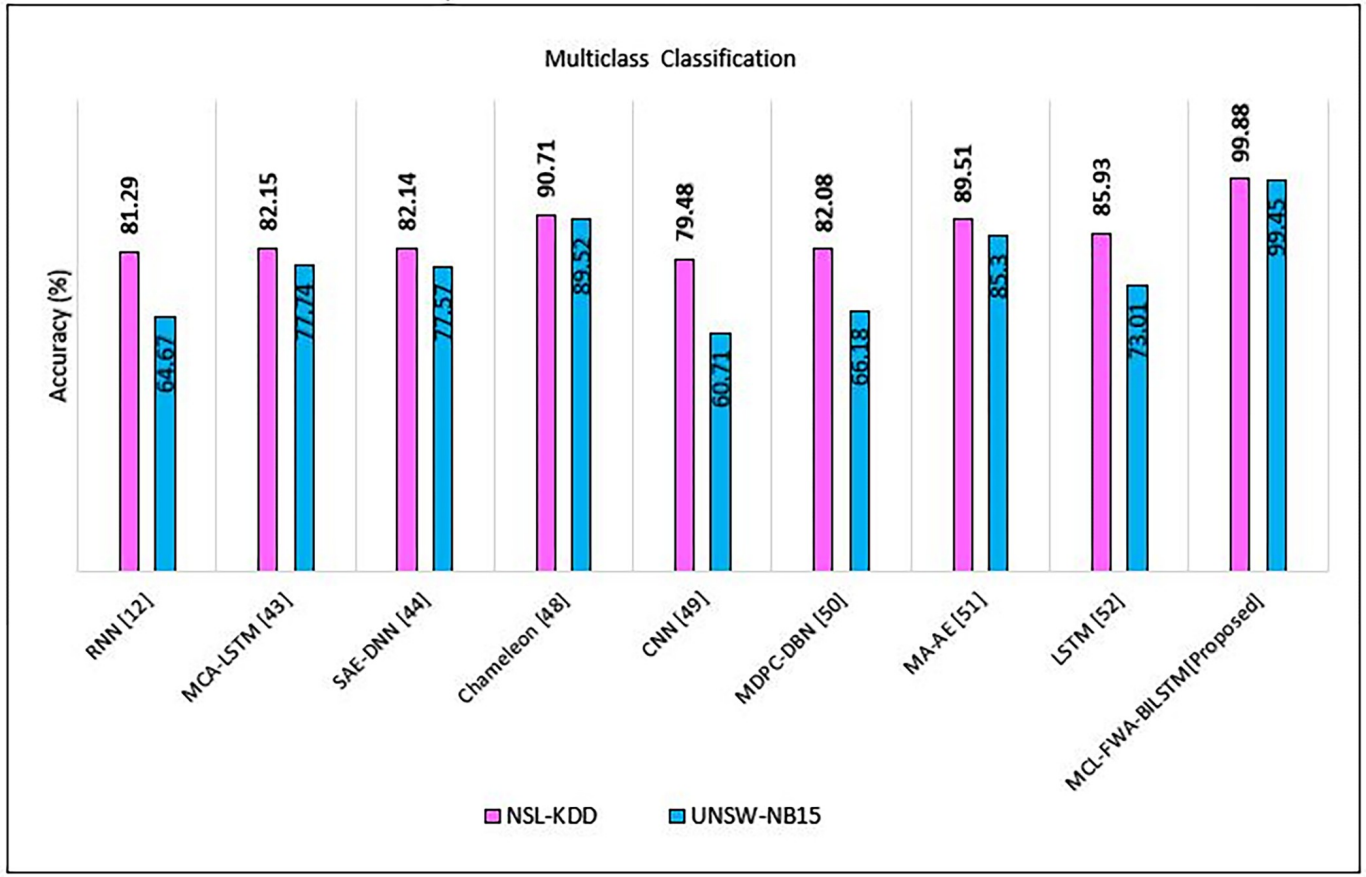
MCL-FWA-BILSTM accuracy comparison with existing approaches for multiclass classification.

**Fig 12:**
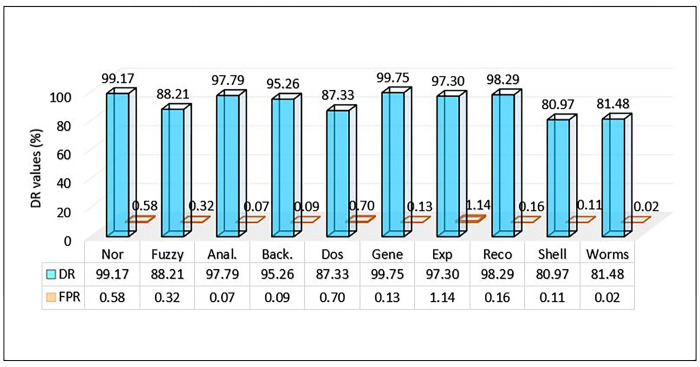
Comparison of DR and FPR of UNSW-NB15.

**Fig 13:**
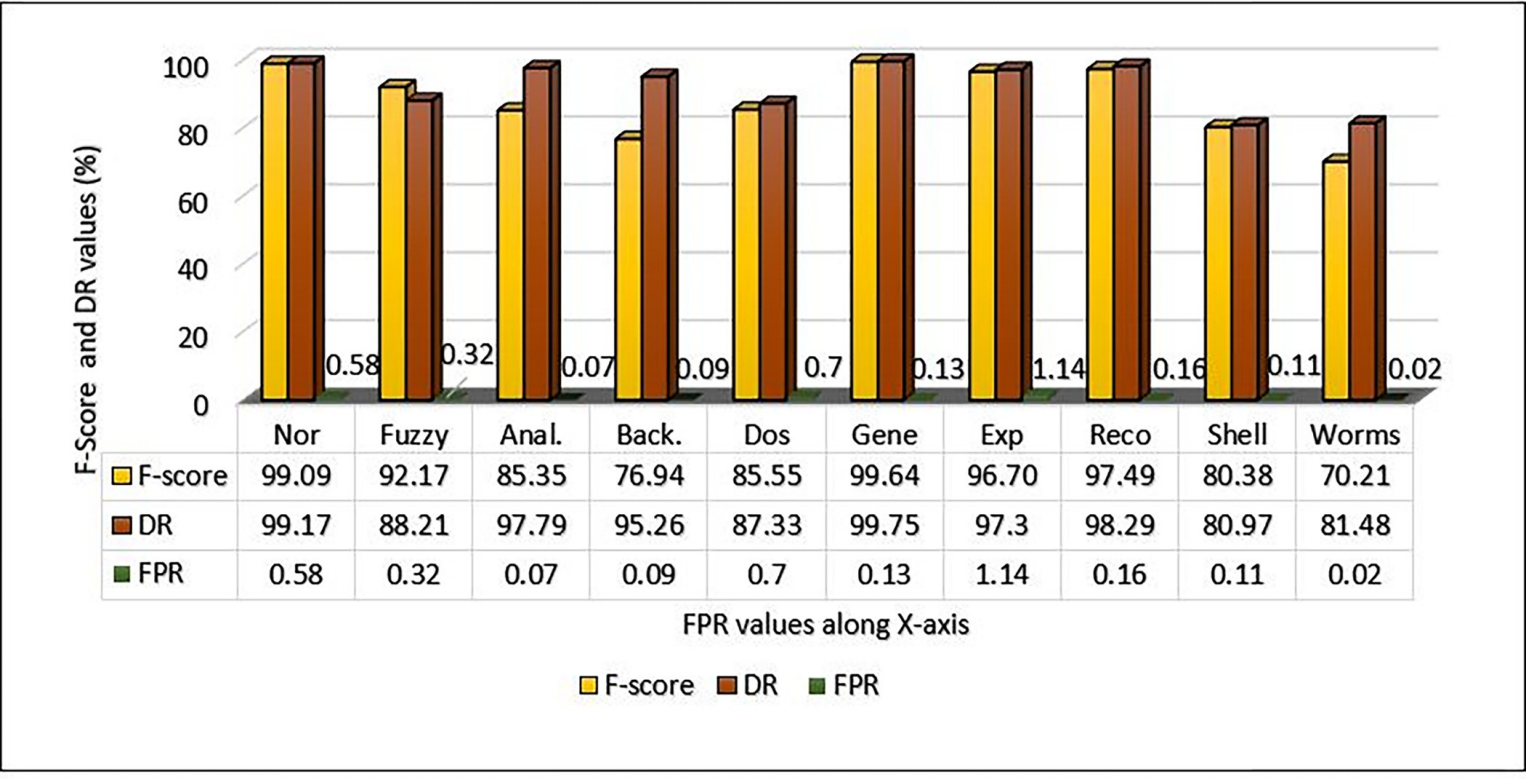
F-Score, DR and FPR comparison for multiclass classification on UNSW-NB15.

**Fig 14:**
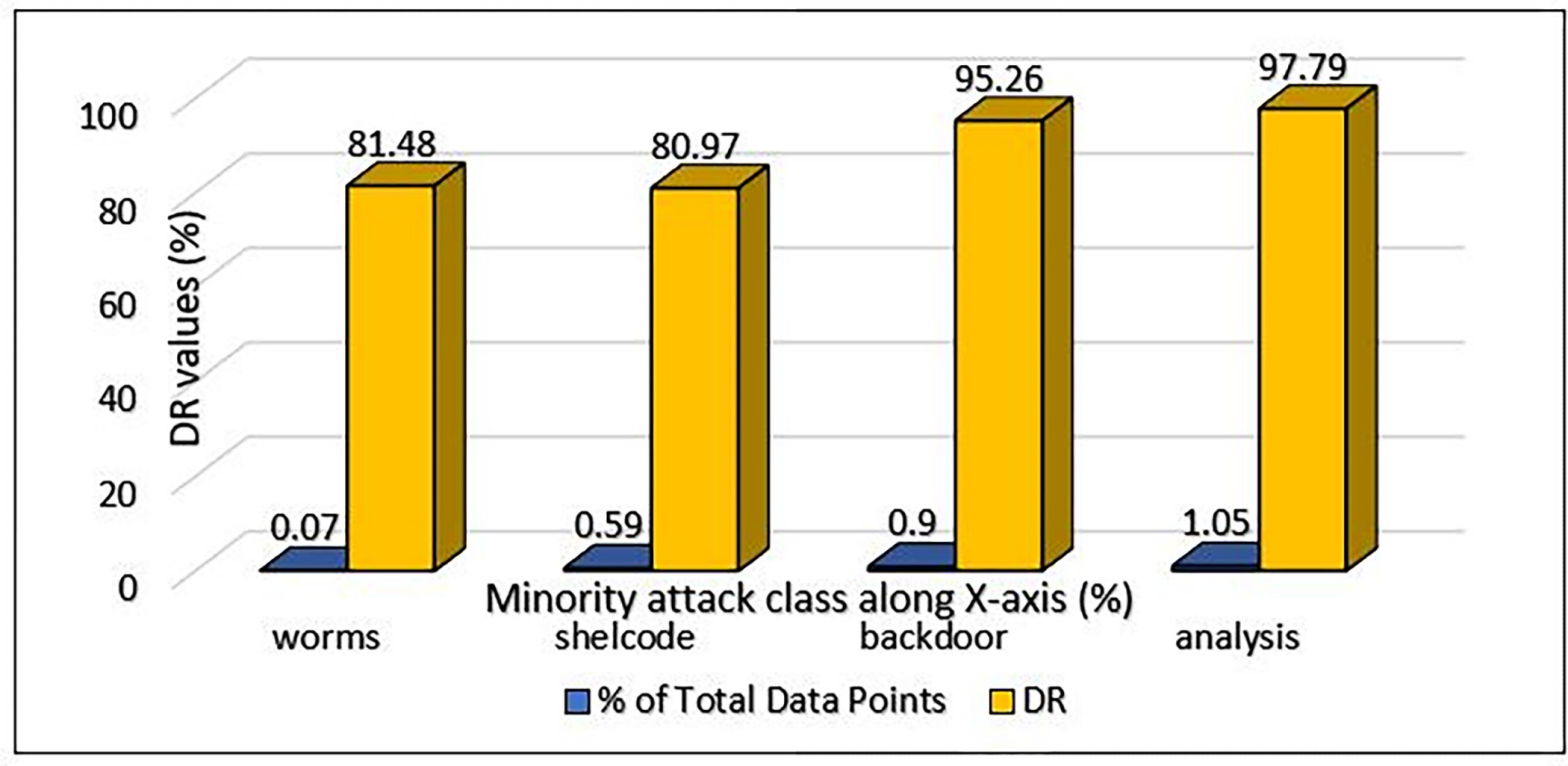
Minority attack class detection rate of UNSW-NB15 using MCL-FWA-BILSTM model.

**Fig 15:**
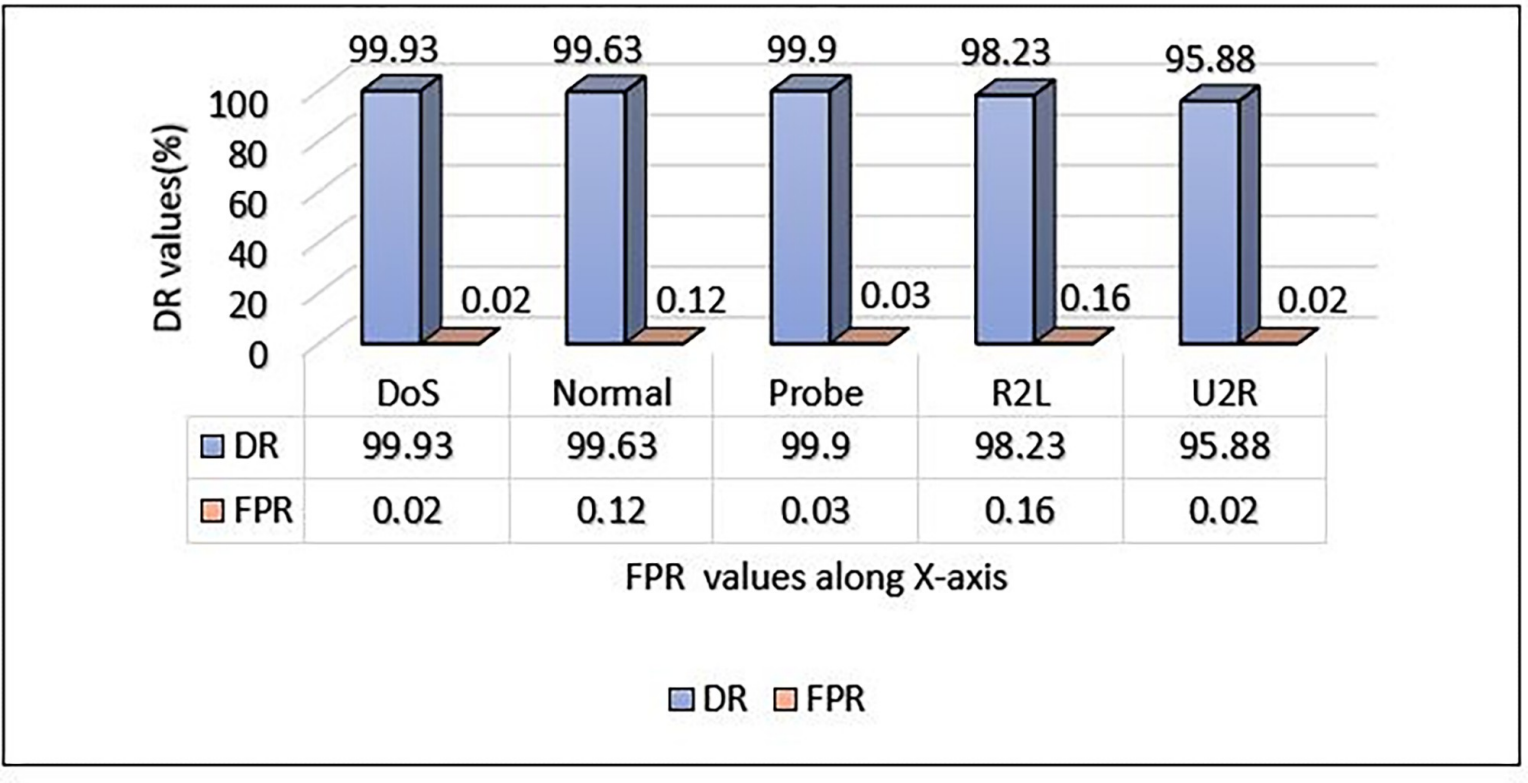
Comparison of DR and FPR of NSL- KDD dataset using MCL-FWA-BILSTM model.

**Fig 16:**
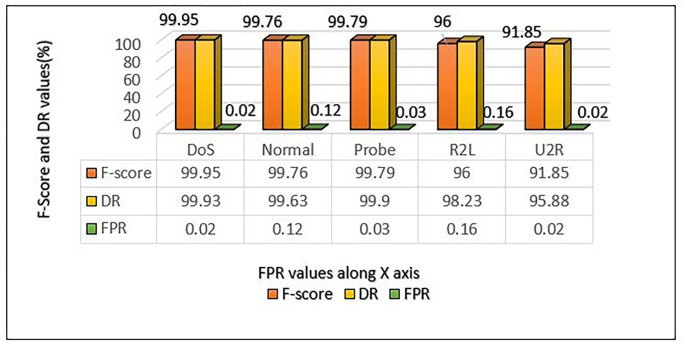
F-score, DR and FPR comparison for multiclass on NSL-KDD using MCL-FWA-BILSTM.

**Fig 17:**
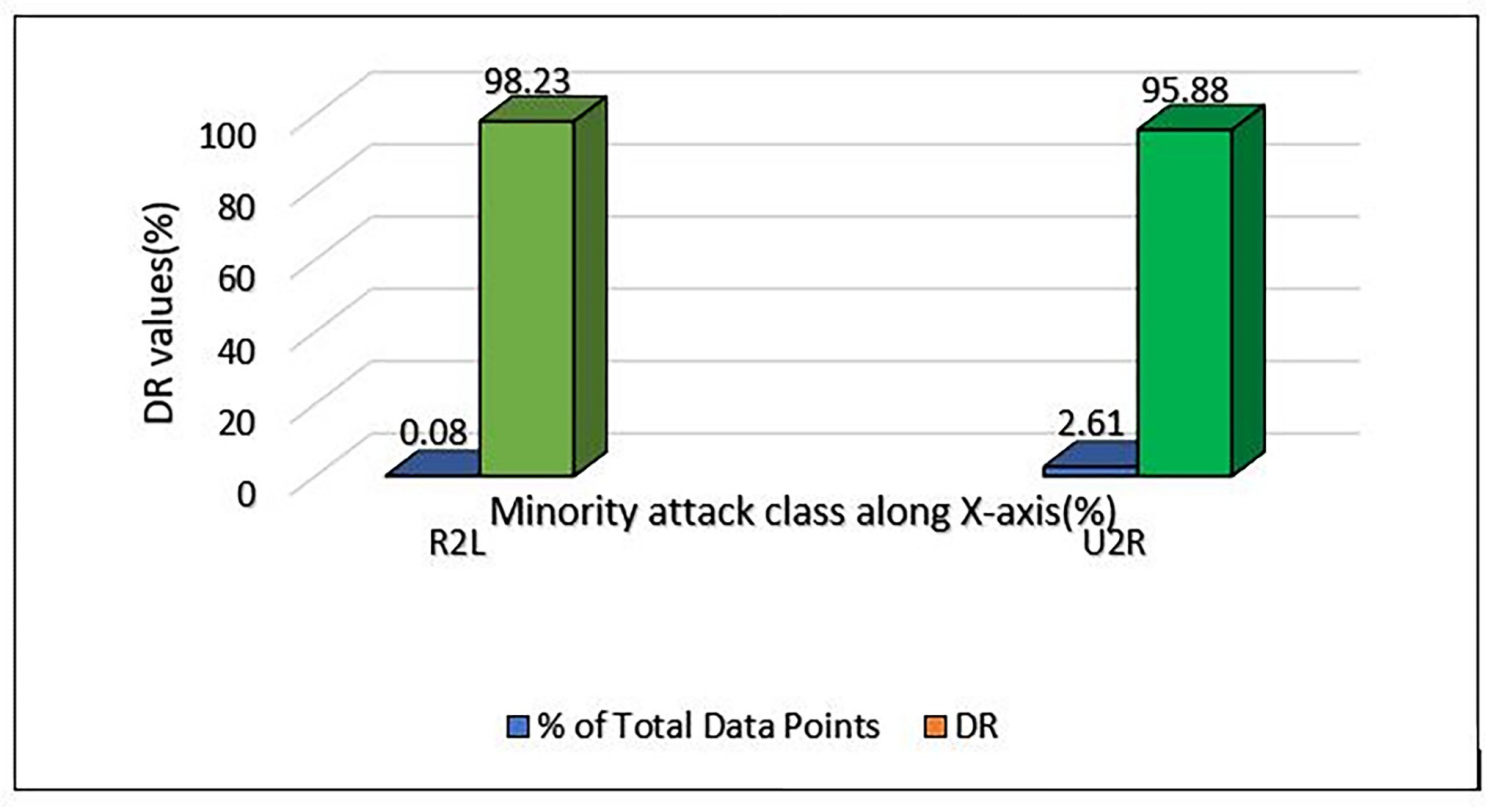
Minority attack class detection rate of NSL-KDD dataset using MCL-FWA-BILSTM model.

**Fig 18:**
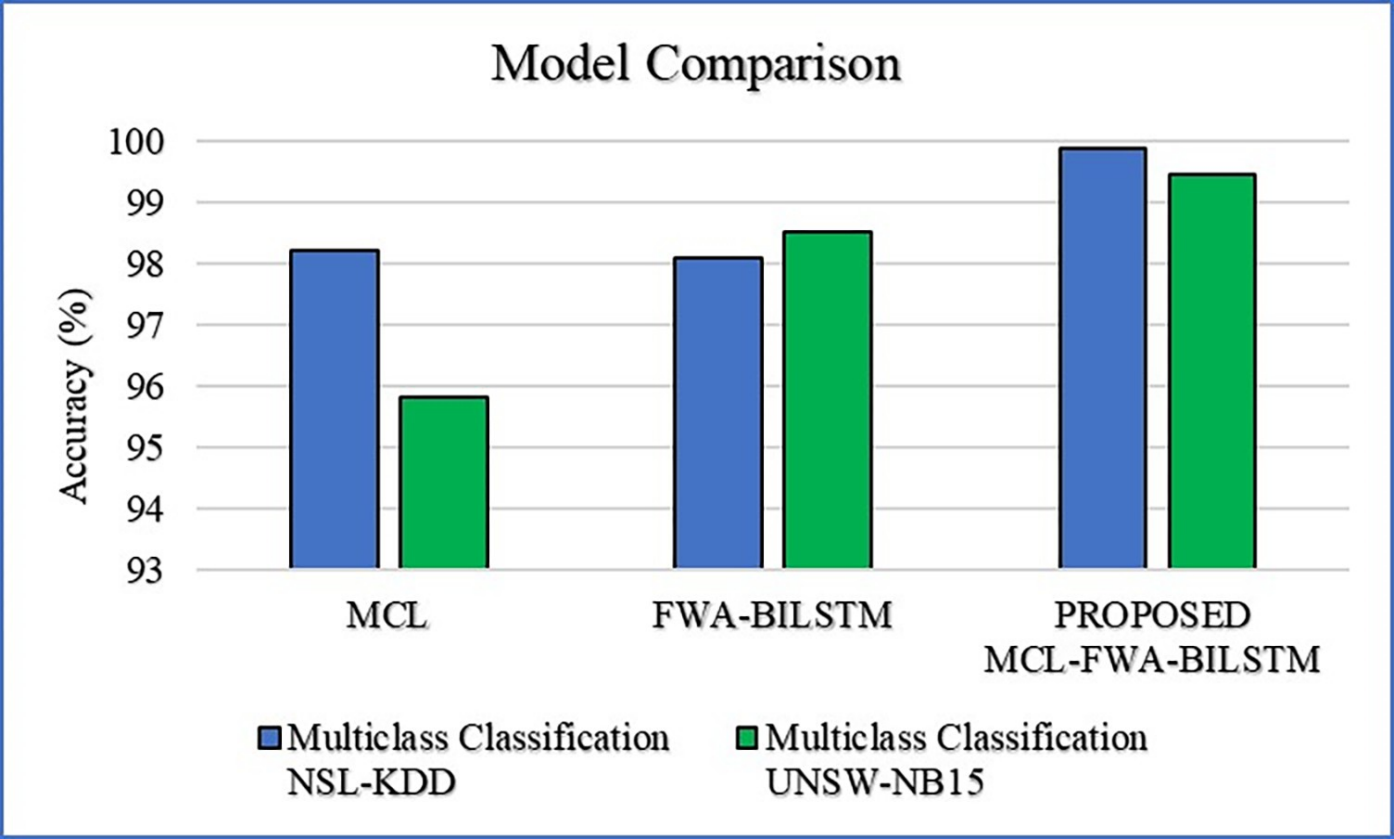
Accuracy measures for MCL, FWA-BILSTM and proposed MCL-FWA-BILSTM.

**Table 8:** Accuracy comparison with existing approaches for Binary Classification with state of art on UNSW-NB15 and NSL-KDD.

Methods	Binary Classification	
	NSL-KDD	UNSW-NB15
Resnet [1]	79.14	81.57
GoogLeNet [1]	77.04	81.84
RNN [12]	83.28	68.55
MCA-LSTM [43]	80.52	88.11
SAE-DNN [44]	87.74	82.97
LSTM [45]	88.13	85.08
Proposed MCL-FWA-BILSTM	99.67	99.56

**Table 9:** Accuracy comparison with existing approaches for binary classification with state of art on UNSW-NB15 and NSL-KDD.

**Methods**	**NSL-KDD**	**UNSW-NB15**
DLNID [24]	90.73	NA
SAE-SVM [46]	84.96	NA
CNN-LSTM [46]	74.77	NA
MCL-FWA-BILSTM[Proposed]	99.67	NA
FFDN [28]	NA	87.1
Smote + GMM [47]	NA	96.54
Chameleon [48]	NA	89.52
MCL-FWA-BILSTM[Proposed]	NA	99.56

**Table 10:** MCL-FWA-BILSTM accuracy comparison with existing approaches for multiclass classification with state of art on UNSW-NB15 and NSL-KDD.

	Multiclass Classification	
Methods	NSL-KDD	UNSW-NB15
RNN [12]	81.29	64.67
MCA-LSTM [43]	82.15	77.74
SAE-DNN [44]	82.14	77.57
Chameleon [48]	90.71	89.52
CNN [49]	79.48	60.71
MDPC-DBN [50]	82.08	66.18
MA-AE [51]	89.51	85.3
LSTM [45]	85.93	73.01
Proposed MCL-FWA-BILSTM	99.88	99.45

**Table 11:** MCL-FWA-BILSTM accuracy comparison with existing approaches for multiclass classification with state of art on UNSW-NB15 or NSL-KDD.

Prior Research Work	NSL-KDD	UNSW-NB15
Autoencoder [2]	84.24	NA
DLNID [24]	90.73	NA
SAE-SVM [52]	80.48	NA
CNN-LSTM [46]	68.78	NA
DNN [53]	75.75	NA
Sem-LSTM [54]	82.21	NA
LSTM [55]	82.78	NA
LSTM [56]	82	NA
CNN+BILSTM [57]	83.58	NA
DLHA [58]	87.55	NA
Proposed MCL-FWA-BILSTM	99.88	NA
FFDNN [28]	NA	77.16
SMOTE + GMM[47]	NA	95.54
CSCADE-ANN[59]	NA	95.98
ICVAE-DNN[60]	NA	89.08
ANN-MLP [61]	NA	76.96
CNN [62]	NA	93.5
J48[63]	NA	87.65
Proposed MCL-FWA-BILSTM	NA	99.45

**Table 12:** a. MCL-FWA-BILSTM comparison with the state of art on UNSW-NB15. **b**. MCL-FWA-BILSTM model DR and FPR achievement.

**Model**	**Adaboost [64]**	**LSTM [65]**	**HAST-IDS [66]**	**SVM [67]**	**MCL-FWA-BILSTM**
DR	91.13	92.06	93.65	83.71	Table 12B
FPR	22.11	4.22	9.6	7.73	Table 12B
Acc.	73.19	92.33	80.03	74.8	99.45

**Category**	**Nor**	**Fuzzy**	**Anal.**	**Back.**	**Dos**	**Gene**	**Exp**	**Reco**	**Shell**	**Worms**
DR	99.17	88.21	97.79	95.26	87.33	99.75	97.3	98.29	80.97	81.48
FPR	0.58	0.32	0.07	0.09	0.7	0.13	1.14	0.16	0.11	0.02

**Table 13:** MCL-FWA-BILSTM comparison with the state of art on NSl-KDD.

Attack Type	RNN [12]		CNN [49]		FSL [68]		MCL-FWA- BILSTM	
	DR	FPR	DR	FPR	DR	FPR	DR	FPR
DoS	83.5	2.1	83.2	2.4	94.1	1.18	99.9	0.02
R2L	24.7	0.8	21.7	0.7	75.9	0.99	98.2	0.03
U2R	11.6	0.1	13	0.1	81.5	0.3	95.9	0.16
Probe	83.4	2.2	81.9	2.1	92.7	1.48	99.9	0.02

### A. Comparison of MCL-FWA-BILSTM model with the state of the art for Binary Classification

To objectively evaluate the accuracy and differentiation of the MCL-FWA-BILSTM model, we compared our model with some recent related works presented by several other researchers, shown in Table 8 and Fig 10, available in open sources. Experimental results confirmed that the proposed approach outperforms most other methods concerning binary classification accuracy. The results showed that the proposed model (99.67%) performed better than other existing techniques in the NSL-KDD datasets. For the UNSW-NB15 dataset, the MCL-FWA-BILSTM model performed optimally (99.56%) in comparison with the existing methods, as shown in Fig 10. The experimental results showed that the performance of MCL-FWA-BILSTM model is superior to that of models based on traditional machine learning methods and other deep learning methods in binary-class classification using the same datasets, NSL-KDD and UNSW-NB15, for network traffic classification. Further, some researchers validated their work using the NSL-KDD benchmark dataset. While few researchers validated their work using the UNSW-NB15 benchmark dataset only. The comparison of all those model results with the proposed hybrid model MCL-FWA-BILSTM visualization is shown in Table 9. It is evident that the proposed model outperformed almost all the previous models.

### B. Comparison with the state of the art for multiclass classification

To assess the accuracy and differentiation of MCL-FWA-BILSTM model, we conducted a comparative analysis with various related works from open-source materials. The experimental findings validate that our approach surpasses most other methods in terms of multiclass classification accuracy for both datasets examined. In particular, MCL-FWA-BILSTM model achieved excellent results (99.88%) on the NSL-KDD dataset compared to other methods, while it did the best (99.45%) on the UNSW-NB15 dataset compared to other methods as shown in Table 10. Previous researchers in this field used the NSL-KDD and UNSW-NB15 datasets to identify network traffic. The obtained results demonstrated that the MCL-FWA-BILSTM model outperformed traditional machine learning models and other deep learning techniques for multi-class classification using these datasets. Fig 11 depicted the results of comparing accuracy across various studies conducted on the NSL-KDD and UNSW-NB15 datasets. Some researchers validated their work using the NSL-KDD benchmark dataset, while others used only UNSW-NB15, the comparison of MCL-FWA-BILSTM model and others shown in Table 11.

### C. DR and FPR comparison for multiclass classification on UNSW-NB15

Fig 12 illustrates the performance of the proposed method on the UNSW-NB15 dataset with respect to the detection rate and FPR. The model obtained an excellent detection rate for Gene, with an FPR of 0.13. Worms have the lowest FPR value, but their detection rate is very low in comparison to most other attacks. As discussed in the above section only a high DR value does not ensure the effectiveness of the model unless its FPR is low. It is evident from the results, as shown in the Fig 12, that the MCL-FWA-BILSTM model achieved the lowest FPR and the highest DR in most of the cases, which confirms the model’s efficiency. The detection rate of each of the ten classes is plotted against the FPR in Fig 12. The highest DR is 99.75% for the generic class, and the lowest DR obtained is 80.97% for the shell category. While the model obtained FPR with a maximum value of 1.14% for exploits and a minimum value of 0.02% for worms. The result obtained by the model MCL-FWA-BILSTM showed that it can classify Nor, Back, DoS, Exp, Shell, Gene, Reco, and Wrm very accurately. The performance of the MCL-FWA-BILSTM model is comparatively better than any state-of-the-art model, as shown in Table 12. The MCL-FWA-BILSTM model achieved best performance in multiclass classification, with an accuracy of 99.45%.

### D. F-score, DR and FPR comparison for multiclass classification on UNSW-NB15using MCL-FWA-BILSTM approach

The F-score for multiclass UNSW-NB15 dataset showed excellent performance above 90% for Normal, Fuzzy, Exploits, Generic, and Reconnaissance as shown in Fig 13. While for analysis, Dos and shellcode the achieved F-score values found above 80%. On the other hand, for the backdoor and worms, the F-score value found below the 80% but the DR value of Backdoor found 95.26% and also the FPR was small as 0.09. Further it is observed from the Fig 13, worms attained the DR value as 81.48% and the FPR as 0.02. So, we can conclude that even for all the 10 classes, MCL-FWA-BILSTM model showed excellent performance concerning the F-score, DR and FPR.

### E. Minority class detection from UNSW-NB15

In UNSW-NB15 dataset, the worms, shellcode, analysis and the backdoor belong to minority traffic. The worms attacks account for 0.07%, shellcode 0.59%, backdoor 0.9%, and the analysis attacks account for 1.05%. Nonetheless, by means of our suggested approach, MCL-FWA-BILSTM, this model managed to identify such attacks with a considerably elevated level of detection rate as shown in Fig 14. It is evident from Fig 14, in relation to analysis attacks, the model attained a highest detection rate of 97.79%, and for the shellcode, the least value of DR was found 80.97%. Moreover, the total accuracy score amounted to an impressive 99.45%. This indicates that the model effectively improved the imbalance issue and the model’s performance.

### F. Comparison of MCL-FWA-BILSTM approach with the state of art considering DR and FPR and Accuracy using UNSW-NB15

It is evident from the comparison as shown in Table 12A and 12B the MCL-FWA-BILSTM model performed exceptionally well on all metrics, including DR, FPR, and accuracy. Further, it is more obvious from the results each category (DR and FPR) shown in Table 12B outperformed all other models. This makes the proposed model preferred for usage in intrusion detection systems.

### G. DR and FPR comparison for NSL-KDD

Fig 15 illustrates the performance of the proposed model on the NSL-KDD dataset with respect to the detection rate and FPR. The model obtained the highest detection rate for DOS and the lowest value for FPR. It is observed experimentally that a model is considered effective if its detection rate(DR) is high and its FPR is low. It is evident from the results, as shown in Fig 15, that the proposed model achieved the lowest FPR, which confirms the model’s robustness and efficiency. On the other hand, the model performance is lowest in terms of DR for the U2R, and the FPR value is comparatively large for the U2R, among others. Examining the plot of class DR and FPR in Fig 15, the best detection rate (DR) is 99.93% for DoS, and the best false positive rate is 0.02% for DoS and U2R. The least DR is 95.88 for U2R, and the highest FPR value is 0.16 for R2L. It is very clear that the model is able to classify all the classes very accurately.

### H. F-score, DR and FPR comparison for multiclass classification on NSL-KDD using MCL-FWA-BILSTM approach

It is evident from the Fig 16, the F-score for multiclass using NSL-KDD dataset, showed excellent performance above 90% for all five classes in terms of F-score as well as DR. Further, FPR value also considerably low for all the five classes. So, we can say the MCL-FWA-BILSTM model achieved excellent classification performance concerning the F-score, DR and FPR related to all attack class of NSL-KDD.

### I. Minority class detection from NSL-KDD

In NSL-KDD, the minority traffic is the R2L and U2R attacks. The R2L attacks account for 0.08%, while the U2R attacks account for 2.61%. Nonetheless, by means of our suggested approach, MCL-FWA-BILSTM, we managed to identify such attacks with a considerably elevated level of detection rate as shown in Fig 17. For example, in relation to R2L attacks, we attained a detection rate of 98%, and for the U2R attempts, this rate was as high as 96%. Moreover, the total accuracy score amounted to an impressive 99.88%. This indicates that the model effectively improved the imbalance issue and the model’s performance.

### J. Comparison of MCL-FWA-BILSTM approach with the state of art considering DR and FPR results on NSL-KDD

We compared the indicators of our method with others state of art models in Table 13. The proposed MCL-FWA-BILSTM method outperformed all other deep learning-based models in terms of DR and also in terms of FPR. Based on the evaluation, results obtained from NSL-KDD dataset, the MCL-FWA-BILSTM model outperformed other approach in nearly all aspects. Specifically, when examining the two categories with adequate samples, we observed that our method’s detection rate surpasses 95%, while other techniques typically exhibit rates below 85%. Regarding the FPR metric, we note that this metric also outperformed all other models except the FPRs of U2R, which are slightly inferior to U2R RNN [12].

### K. Comparison of MCL-FWA-BILSTM approach with the existing MCL and FWA-BILSTM model

In the Fig 18, bar chart illustrates a comparative analysis of three different models’ performance on multiclass classification tasks across two datasets, NSL-KDD and UNSW-NB15. The vertical axis represents classification accuracy percentages, while the horizontal axis identifies the models: MCL [69], FWA-BILSTM [70], and proposed MCL-FWA-BILSTM method. For the NSL-KDD dataset, MCL [69] shows lower performance, while the proposed MCL-FWA-BILSTM method achieves the highest accuracy, closely followed by FWA-BILSTM. In contrast, for the UNSW-NB15 dataset, the proposed MCL-FWA-BILSTM method still leads with the highest accuracy, but it shows a significant improvement over its performance on NSL-KDD, surpassing FWA-BILSTM. This visual representation emphasizes the superiority of the proposed MCL-FWA-BILSTM method in multiclass classification accuracy for both datasets.

Based on the outcomes, it is evident that the MCL-FWA-BILSTM hybrid deep learning model outperformed other existing techniques owing to its superior performance. In fact, it utilized the novel hybrid mechanism to enhance the classification performance. When utilizing the UNSW-NB15 dataset, the proposed model achieved the highest accuracies of 99.56% and 99.45% in the cases of binary and multiclass classification, respectively. Also, for the NSL-KDD, the achieved accuracies for binary and multiclass are 99.67% and 99.88%, respectively. Further, this model showed enhanced DR and FPR values in comparison to other deep learning machine learning methods as shown in Tables 12 and 13.

### L. Reason for the improved results

In intrusion detection, primary challenge data or situations of class imbalance are detected, and in real-time, new attacks that have not been detected previously are detected; how can this be resolved? Although the results indicate improvement, the reason for this improvement is discussed in this section. The proposed approach involves the transmission of features via two networks. The first is the attention mechanism. Layers of attention refine the features based on their relative weight. The second section uses BI-LSTM to decide how to use learning features and how much weight to give them based on semantic relationships. In both processes, the proposed approach obtains the most efficient weight of components and aids in parameter learning. During training, learning parameters determines whether a feature is significant and should receive domain or class weights. If so, the learning phase using ensemble learning improves. So far, this section has talked about why learning gets better. Now we will discuss the impact of our new algorithm on challenges. First, we deal with the class imbalance; our algorithm enhances learning in the presence of class imbalance due to the semantic weight of its features. If we have some instances, it is capable of detecting additional instances. The second challenge in real-time is that if a new sample arrives, the attention mechanism must be capable of performing a weighted attack rather than an attack on the class. So, it will be easy for the mechanism to figure out its binary type.

### M. Statistical analysis

Table 14, based on nine approaches or measurements presents descriptive statistics for two datasets–NSLKDD and UNSWNB15. Both of these mean figures reflect the average value acquired from the nine approaches. NSLKDD has a mean of about 85.91, which is higher than that of UNSWNB15, which has a mean of around 77.13, smaller std. err. (Stand Error) indicate high precision in representing a sample’s average as the population’s one. In relation to UNSWNB15, it is essential to note that NSLKDD has a standard error of about 2.16, while for UNSWNB15, it is about 4.22, thus indicating a more precise estimation of its MEAN. The 95% and 99.9% confidence intervals(CI) give an estimate within which the true mean of the population will lie, assuming we have taken a representative sample. NSLKDD shows both a higher mean and a more precise estimation of that mean (as indicated by the more minor standard error and narrower confidence intervals). The interval between 80.92 and 90.90 contains the actual population mean for NSLKDD with a relatively confident level of likelihood, though with significant probability errors. In contrast, in the case of UNSWNB15, this interval holds between 67.40 and 86.85 because these CIs are more extensive due to big standard errors. The wider range interval (55.87–98.39) accounts for 0.01% margin error at most for UNWNB15’s estimated means with such a high degree certainty level as usually happens when raising confidence level beyond lower limits where narrower ones can be realized. Moreover, it should be noted that when compared to other measures, NSLKDD demonstrates broader ranges due to more minor standard errors. Thus, we can say that if the data completeness approach is used in evaluation studies, then NSLKDD would yield better results than KDDCUP99.

**Table 14:** MCL-FWA-BILSTM and other existing approaches for multiclass classification in both datasets.

Variable	Approaches	Mean Std. err.	[95% conf. interval]	[99.9% conf. interval]
NSLKDD	9	85.90778 2.163042	80.91979 90.89576	75.00322 96.81233
UNSWNB15	9	77.12778 4.217612	67.40195 86.85361	55.86551 98.39005

## V. Conclusion

Current research on the application of deep learning approaches for network traffic classification has not fully utilized the structured information inherent in network traffic. We implemented a unique hybrid model termed MCL-FWA-BILSTM, which combines a Bidirectional Long Short-Term Memory (BILSTM) model with Sentiment Analysis (SA) trained with Random Forest learning. This strategy utilizes deep learning techniques for applications in Machine Learning (ML). The proposed approach utilizes feature weighting to enhance the domain or pattern knowledge provided to the classifier. The FWA-BILSTM part of the approach provides a higher weight to the unique features that helps to distinguish the changes in the traffic. As a result, the classifier learns efficiently and distinguishes between attacks and normal class means while learning different types of attacks. The results of this process improve significantly when a less complex proposed model is used. In multiclass datasets, classes with few instances were enhanced by 6 to 10%, while classes with a large number of instances improved by 1–2%, indicating that we improved the main challenge class imbalance in the two classes by 0.8 to 0.9 percent. Additionally, our approach is significant because it enhances the real-time detection of attacker classes.

The main limitation of the proposed research work is that it needs to improve the high-level class imbalance present in other intrusion network datasets. So, future work will be based on different datasets that could be more balanced. The second limitation of the proposed work is that it will improve the accuracy of high imbalance data in multiclass classification. So, in future enhancements, improve the model and make it more generalized.

## Supporting information

S1 File Supporting Info File NSL KDD. UNSW-NB15 dataset can be downloaded from https://research.unsw.edu.au/projects/unsw-nb15-dataset.
(ZIP)
